# Celastrol-regulated gut microbiota and bile acid metabolism alleviate hepatocellular carcinoma proliferation by regulating the interaction between FXR and RXRα *in vivo* and *in vitro*

**DOI:** 10.3389/fphar.2023.1124240

**Published:** 2023-02-15

**Authors:** Dequan Zeng, Lipen Zhang, Qiang Luo

**Affiliations:** ^1^ Key Laboratory of Design and Assembly of Functional Nanostructures, Fujian Institute of Research on the Structure of Matter, Chinese Academy of Sciences, Fuzhou, China; ^2^ Department of Translational Medicine, Xiamen Institute of Rare Earth Materials, Chinese Academy of Sciences, Xiamen, China; ^3^ School of Pharmaceutical Science, Xiamen University, Xiamen, China

**Keywords:** celastrol, hepatocellular carcinoma, *Bacteroides fragilis*, glycoursodeoxycholic acid, farnesoid X receptor

## Abstract

Celastrol, a triterpene derived from *Thunder God Vine* (*Tripterygium wilfordii Hook f*; Celastraceae), a traditional Chinese herb, has promising anticancer activity. The present study aimed to elucidate an indirect mechanism of celastrol-mediated alleviation of hepatocellular carcinoma (HCC) *via* gut microbiota-regulated bile acid metabolism and downstream signaling. Here, we constructed a rat model of orthotopic HCC and performed 16S rDNA sequencing and UPLC-MS analysis. The results showed that celastrol could regulate gut bacteria; suppress the abundance of *Bacteroides fragilis*; raise the levels of glycoursodeoxycholic acid (GUDCA), a bile acid; and alleviate HCC. We found that GUDCA suppressed cellular proliferation and induced the arrest of mTOR/S6K1 pathway-associated cell cycle G0/G1 phase in HepG2 cells. Further analyses using molecular simulations, Co-IP, and immunofluorescence assays revealed that GUDCA binds to farnesoid X receptor (FXR) and regulates the interaction of FXR with retinoid X receptor *a* (RXRα). Transfection experiments using the FXR mutant confirmed that FXR is essential for GUCDA-mediated suppression of HCC cellular proliferation. Finally, animal experiments showed that the treatment with the combination of celastrol/GUDCA alleviated the adverse effects of celastrol alone treatment on body weight loss and improved survival in rats with HCC. In conclusion, the findings of this study suggest that celastrol exerts an alleviating effect on HCC, in part *via* regulation of the *B. fragilis*-GUDCA-FXR/RXRα-mTOR axis.

## 1 Introduction

Celastrol is a natural pentacyclic triterpene derived from the root of *Thunder God Vine*, a traditional Chinese medicinal plant (Ng et al., 2019; Chen et al., 2018). Celastrol is considered as one of the most potential five natural medicinal compounds (others include artemisinin, triptolide, capsaicin, and curcumin) (Corson and Crews, 2007). The extracts of *Thunder God Vine* have been shown to exert hepato-protective effects and have been used to alleviate liver injury (Qi et al., 2020; Yan et al., 2021), hepatic inflammation (Luo et al., 2017), and hepatocellular carcinoma (HCC) (Chang et al., 2016). The direct effect on the signaling process in hepatocytes is considered the main molecular mechanism of celastrol-mediated alleviation of hepatic diseases. Our previous study revealed that celastrol could repair acute liver injury by directly targeting the nuclear receptor Nur77 in the liver tissue and clearing inflamed mitochondria (Hu et al., 2017). Furthermore, our recent finding also showed that celastrol regulated fecal morphology and changed the gut microbiota community structure during alleviating HCC proliferation in an orthotopic HCC rat model. Emerging evidence shows that gut microbiota regulates the progress of carcinoma, wherein celastrol has been shown to regulate gut microbiota to maintain the immune balance of ulcerative colitis and inhibit lipid absorption in obesity. These studies imply that the gut microbiota may, in part, contribute to the HCC-alleviating effect of celastrol.

As an important micro-ecosystem of the human body, gut microbiota affects liver bile acid metabolism *via* enterohepatic circulation (Chávez-Talavera et al., 2017; Staley et al., 2017) and regulates the development of hepatic diseases such as hepatitis (Li et al., 2020), cirrhosis (Ridlon et al., 2015; Kakiyama et al., 2013), and HCC (Yamada et al., 2018). Gut microbiota usually adheres to the intestinal mucosa or excretes with feces, making it difficult to enter the liver. However, the occurrence of unbalanced gut homeostasis with changes in microbiota community structure and an increase in the abundance of pathogenic bacteria damage the intestinal mucosa, leads to the migration of the microorganisms to the liver, affects the metabolism of bile acids, and eventually leads to the development of hepatic diseases, such as HCC. Recently, several studies have revealed that gut microbiota and liver bile acid metabolism regulation are involved in HCC progression (Yamada et al., 2018; Wu et al., 2021; Chiang and JessicaFerrell, 2018). Additionally, bile acid is an important endogenous active molecule regulating liver function and exerts its function *via* binding to the farnesoid X receptor (FXR) and regulating the heterodimer of FXR (Chiang and JessicaFerrell, 2020; Radun and Trauner, 2021; Molinaro and Marschall, 2022). Therefore, we speculated that exploring the mechanism underlying the regulation of gut microbiota associated with liver bile metabolism, regulation of binding to FXR, the heterodimer of FXR, and HCC proliferation could contribute to the understanding of the alleviating effect of celastrol against HCC.

To test this hypothesis, we constructed a rat model of orthotopic HCC induced by diethyl nitrosamine (DEN) and evaluated the effect of celastrol on HCC in this study. We performed 16S rDNA sequencing of rat feces to identify potential active bacterial species and UPLC-MS analysis of liver tissue to identify active bile acids. The effect of active bile acid on the proliferation of hepatoma carcinoma HepG2 cells was confirmed using MTT assay and clone formation. We also evaluated the mTOR/S6K1 proliferation pathway and cell cycle distribution using western blotting and flow cytometry, respectively. Molecular simulations and SPR assays were performed to evaluate the binding of active bile acid to FXR, and a reporter assay was employed to study the effect of active bile acid on the transcriptional activity of FXR. Co-IP and immunofluorescence assays were performed to elucidate the effect of active bile acid on the heterodimer of FXR with retinoid X receptor *a* (RXRα), and transfection experiments with the FXR mutant were performed to elucidate the role of FXR in active bile acid-regulating cellular proliferation and affecting mTOR/S6K1 pathway-related cell cycle distribution. Finally, a rat model with orthotopic HCC was used to evaluate the synergistic effect of the active bile acid/celastrol combination and to analyze the effect difference between the active bile acid/celastrol combination and celastrol alone.

## 2 Materials and methods

### 2.1 Materials

The materials used in this study are listed in Table 1.

**TABLE 1:** The materials used in this study.

Materials	Source	Identifier
Antibodies		
Anti-mTOR	Abcam, Cambridge, UK	Ab32028
Anti-p-mTOR	Abcam, Cambridge, UK	Ab109268
Anti-S6K1	Abcam, Cambridge, UK	Ab32529
Anti-p-S6K1	Abcam, Cambridge, UK	Ab59208
Anti-p-Rb	Abcam, Cambridge, UK	Ab184796
Anti-myc	Abcam, Cambridge, UK	ab9106
Anti-flag	Abcam, Cambridge, UK	Ab205606
Anti-FXR	Abcam, Cambridge, UK	Ab129089
Anti-cyclin D1	CST, Boston, United States	55,506
Anti-CDK4	CST, Boston, United States	3,136
Anti-CDK6	Abcam, Cambridge, UK	AB151247
Anti-cdc25A	Abcam, Cambridge, UK	Ab79252
Anti-p21	Abcam, Cambridge, UK	Ab109520
Anti-ki67	Abcam, Cambridge, UK	Ab16667
Anti-β-actin	Abcam, Cambridge, UK	Ab8226
Goat anti-mouse lgG Secondary antibody, HRP conjugate	Abcam, Cambridge, UK	Ab205719
Goat anti-rabbit lgG Secondary antibody, HRP conjugate	Abcam, Cambridge, UK	Ab205718
Chemical and Reagents		
DEN (Diethyl nitrosamine)	Sigma, Saint Louis, UK	N0756
ALT assay kit	Nanjingjiancheng, Nanjing, China	C009-2-1
AST assay kit	Nanjingjiancheng, Nanjing, China	C010-2-1
MTT (methyl thiazolyl tetrazolium)	Abcam, Cambridge, UK	Ab211091
Fecal DNA Isolation Kit	Vazyme, Nanjing, China	DC103
SYBR green dye	Vazyme, Nanjing, China	Q131
Giemsa dye	Solarbio, Beijing, China	G8220
PI (Propidium Iodide)	Solarbio, Beijing, China	P8080
ECL detecting kit	Thermo, Waltham, United States	32,109
BCA protein assay kit	Solarbio, Beijing, China	PC0020
DAPI dye	Solarbio, Beijing, China	C0060
Protein Ladder	Thermo, Waltham, United States	26,616
Protein G Agarose	Thermo, Waltham, United States	15920010
DMEM culture medium	Gibco, New York, United States	11965092
Fetal bovine serum	Gibco, New York, United States	12484028
Eosin dye	Solarbio, Beijing, China	G1100
Hematoxylin dye	Solarbio, Beijing, China	H8070
Transfection reagents	Thermo, Waltham, United States	11668019
Dual-Luciferase reporter assay system	Promega, Madison, United States	E1910
CM5 chip	GE Healthcare Pittsburgh, United States	BR100530
Experimental Models		
HEK 293T cell lines	Cell bank, Shanghai Cell Biology Institute, Shanghai, China	N/A
HepG2 cell lines	Cell bank, Shanghai Cell Biology Institute, Shanghai, China	N/A
Rat	Xiamen University Laboratory Animal Center, Xiamen, China	N/A
Software		
Autodock	MGL	AutoDock 4
PyMol	DeLano Scientific LLC	PyMol V2.2.0
ImageJ	NIH, United States	ImageJ V2.3.0
Prism GraphPad	Insightful Science, United States	GraphPad5
Instruments		
Paraffin Embedding Center	Leica, Germany	EG1150C
Slicer	Leica, Germany	RM2235
Microplate reader	Cmax Plus, United States	Molecular Devices Cmax Plus
Protein Detecting System	Bio-Rad, United States	1645050
Multimode reader	BioTek, United States	Cytation 5
Cell incubator	ESCO, Singapore	CLM-170B-8-NF
Microscope	Motic Electric, China	AE2000
Laser scanning confocal microscope	Carl Zeiss, Germany	LSM880
Flow cytometry	Beckman, United States	CytoFLEX
Centrifuge	Scilogex, China	D3024R

### 2.2 Animal experiments and sampling

Sprague-Dawley rats (6–8 weeks old) were purchased from Xiamen University Laboratory Animal Center (Xiamen, China). After acclimation for 1 week in a controlled atmosphere of 12 h light/dark cycle at 22°C, rats were randomly divided into two groups: normal (*n* = 10) and model (n = 30). Rats in the model group were treated by gavage with DEN at a dose of 10 mg/kg body weight once a week for 3 months, and those in the normal group were treated with normal saline as a control. The DEN solution was prepared by dissolving 1 g DEN in 100 mL normal saline to a final concentration of 10 mg/mL. The rats in the model group were randomly divided into three subgroups: model, celastrol, and celastrol/GUDCA (n = 10/group). Rats in normal and model groups were gavaged with normal saline with 1% DMSO and 5% tween-80 six times per week for 10 weeks, wherein the rats in celastrol and celastrol/GUDCA groups were treated (*via* gavage) with celastrol at 0.5 mg/kg body weight and celastrol/GUDCA at 0.5 mg/kg +20 mg/kg body weight, respectively, 6 times a week for 10 weeks. The celastrol solution was prepared by dissolving 25 mg celastrol in 1 mL DMSO and following diluting with 99 mL normal saline to a final concentration of 0.25 mg/mL. The body weights of the rats were measured once a week, and the survival number was recorded in real-time. At the end of the 10th week of treatment, blood samples were collected from the eyes to estimate serum ALT and AST levels. Fecal samples were collected for extraction of DNA for gut microbiota community analysis. Subsequently, the rats were sacrificed and liver samples were collected for pathological examination and protein expression analysis. All animal experiments were approved by the Animal Care and Use Committee of Xiamen University.

### 2.3 Tissue processing and histological analysis

The liver tissues of the rats were collected and cut into approximate 8.0 mm × 8.0 mm squares, put into 4% paraformaldehyde phosphate buffer solution, and fixed overnight at 4°C. The fixed tissues were then embedded in paraffin and sliced into 4 μm-thick sections. For hematoxylin and eosin (H&E) staining, the sections were deparaffinized using a gradient xylene-alcohol-distilled water solution, stained with H&E stain, and dehydrated using the gradient alcohol-xylene solution. Afterward, the tissue sections were mounted on slides, and images were acquired using a microscope, the images were then analyzed using ImageJ software. To assess the expression of ki67, immunohistochemical analyses were performed. The sections were deparaffinized using the gradient xylene-alcohol-distilled water solution, which were then retrieved using citrate solution (10 mM sodium citrate, pH 6.0) and blocked using 10% goat serum. Afterward, the sections were incubated with a primary antibody of ki67, stained with DAB and hematoxylin, and dehydrated with the gradient alcohol-xylene solution. The sections were then mounted on slides for imaging using a fluorescence microscope and analyzed using ImageJ software.

### 2.4 Estimation of the levels of serum biochemical indicators

The blood samples were collected from the eyes of rats and allowed to stand at room temperature (20°C–25°C) for 30 min, centrifuged at 3,000 rpm (centrifugal radius: 7 cm) for 20 min, and the supernatants were transferred to fresh 1.5 mL tubes. Then the levels of glutamic-oxaloacetic transaminase (AST) and glutamic-pyruvic transaminase (ALT) in the supernatants were detected using micro-plate methods according to the serum biochemical indicator assay kits (Nanjingjiancheng, China) following the manufacturer’s protocol.

### 2.5 Western blotting

For tissue samples, 100 mg liver was cut into pieces on ice, ground into powder in liquid nitrogen, and lysed with 500 μL RIPA lysis buffer containing protease and phosphatase inhibitors. For cell samples, approximately 1 × 10^6^ HepG2 cells per well of 6-wells plate were collected and added 100 μL RIPA lysis buffer containing protease and phosphatase inhibitors. All samples were lysed for 30 min on ice; afterward, the lysis solution was centrifugated at 12,000 rpm and 4°C for 10 min. The supernatants were collected to estimate total protein concentration using a BCA protein assay kit. The samples were diluted to a final concentration of 2 μg/μL and boiled at 105°C for protein denaturation. The denatured protein samples (20 μL) were loaded onto an 8%–12% SDS-PAGE gel for electrophoresis and transferred to a PVDF membrane. The membrane was blocked with 5% defatted milk powder in TBST solution containing tween-20. Afterward, the membrane was incubated with the primary antibodies against mTOR, p-mTOR, S6K1, p-S6K1, p-Rb, myc, flag, FXR, RXRα, cyclin D1, CDK4, CDK6, cdc25A, p21, ki67, and β-actin, followed by incubation with the corresponding secondary antibodies. All primary antibodies were diluted to 1:1,000 or 1:2,000, and secondary antibodies were diluted to 1:10,000. After incubation, the PVDF membranes were visualized using an ECL solution and imaged on the films *via* exposure. The images in the film were quantified for the gray value using ImageJ.

### 2.6 16S rDNA sequencing

The fecal samples were collected and transferred to Xiamen Treatgut Bio-Technology Co., Ltd. for 16S rDNA sequencing and the analysis of the gut microbiota community. In brief, the total DNA of fecal samples was extracted using a fecal DNA isolation kit. The concentration and purity of DNA were quantified using a Multiskan GO microplate reader (Thermo, United States), and the integrity of DNA was detected by agarose gel electrophoresis. After quality testing, the library was constructed using primers 341F (sequence: CCTACGGGNGGCWGCAG) and 806R (sequence: GGACTACHVGGGTATCTA AT) to amplify the 16S v3–v4 regions. The concentration of the library was quantified using Qubit 3.0, the integrity of the library was detected using an Agilent 2,100 bioanalyzer (Agilent, United States), and qPCR was performed to confirm and obtain accurate quantification. After the library was quantified, different libraries were pooled to flow cells and sequenced using a high-throughput sequencer (Illumina). Subsequently, various bioinformatic analyses were performed, including OTU cluster, α-diversity, PCA, cluster heatmap, species difference, and related metabolism.

### 2.7 Estimation of bile acid contents

Liver tissue samples (200 μg) were collected, cut into pieces on ice, ground into powder in liquid nitrogen, and extracted with 2 mL HPLC-grade chloroform/methyl alcohol solution. The extracted solution was centrifuged at 12,000 rpm at 4°C, and the supernatant was harvested and dried under vacuum. The dry bile acid was dissolved in HPLC-grade methyl alcohol, and chlorpropamide was added as an internal standard for quantifying bile acid content. The bile acid samples containing the internal standard were used to assess data from 50 to 800 m/z using an Easy-nLC1000 UPLC-Q Exactive MS system (Thermo, United States). The standards of the bile acids tested in the present study were used to identify the UPLC-MS data.

### 2.8 Cells culture

Human embryonic kidney HEK-293T and human hepatoma HepG2 cells were maintained in DMEM supplemented with 10% fetal bovine serum and 1% penicillin-streptomycin solution. HEK-293T cells were used to detect the effects of GUDCA on FXR transcriptional activity using a reporter assay. Before testing, cells were seeded into 48-well plates, transfected with two plasmids, pBind FXR LBD and pGL5 Luc, and treated with CDCA and GUDCA. HepG2 cells were used to detect the effect of GUDCA on cellular proliferation and the interaction between FXR and RXRα. Before the tests, the cells were seeded into the responding multi-well plate, such as 96-well plates for MTT assay, 6-well plates for western blotting, 100 mm plates for Co-IP assay, and 24-well plates for immunofluorescence assay, and then treated with 50 or 100 μM GUDCA for the corresponding times.

### 2.9 MTT assay

HepG2 cells treated with 50 or 100 μM GUDCA for 48 h were replaced with fresh DMEM medium containing 0.5 mg/mL MTT and incubated in a CO_2_ incubator for 4 h. GUDCA stock was prepared by dissolving 45 mg GUDCA in 1 mL DMSO and MTT stock was prepared by dissolving 100 mg MTT in 20 mL PBS. The stock solutions were filtrated through a 0.22 μm microporous filter. After incubation, the cellular supernatant was removed and replaced with 100 μL DMSO to dissolve the precipitate. The optical density (OD) of the dissolving solution was read at a wavelength of 492 nm using a microplate reader (Thermo, United States).

### 2.10 Cell clone formation

HepG2 cells treated with 50 or 100 μM GUDCA for 10 days were washed twice with PBS and fixed with 3 mL methyl alcohol for 15 min. Then, the methyl alcohol was removed, and the cells were washed twice with PBS and stained with 2 mL Giemsa dye for 30 min. After staining, clones were washed with distilled water and imaged using a camera (Canon, Japan).

### 2.11 Flow cytometry to detect cell cycle distribution

HepG2 cells treated with 50 or 100 μM GUDCA for 24 h were washed twice with PBS and digested with 0.25% trypsin to the single cells. The cells were harvested *via* centrifugation at 1,500 rpm (centrifugal radius: 7 cm) for 10 min, resuspended in pre-cooled 70% alcohol, fixed overnight at −20°C, and stained with a solution containing 50 μg/mL PI and 1 mg/mL RNase A at room temperature in the dark for 30 min. The stained cells were subjected to flow cytometry (Beckman, United States), and the cell cycle distribution was determined.

### 2.12 Molecular simulation

Molecular simulations were performed as described previously (Hu et al., 2017; Luo et al., 2016). Briefly, docking of GUDCA to FXR (PDB ID:3DCT) was performed using AutoDock software, and molecular visualization was displayed using PyMOL software.

### 2.13 Transfection experiment

For the reporter assay in HEK-293T cells, plasmids containing 100 ng pBind FXR LBD and 200 ng pGL5 luc were added to 50 μL Opti-MEM medium as solution A, and 1.5 μL lipofectamine 3,000 reagent was added to 50 μL Opti-MEM medium as solution B. After incubation of solutions A and B for 5 min, the two solutions were mixed and incubated for another 20 min, and then the mixture was gently added to the cells with 150 μL DMEM medium. The cells were maintained for 12 h, and the medium was replaced with normal DMEM. For HepG2 cells, the plasmid containing 1 μg flag-FXR and RXRα was added to 500 μL Opti-MEM medium as solution A, and 15 μL lipofectamine 3,000 reagent was added to 500 μL Opti-MEM medium as solution B. After incubation of solutions A and B for 5 min, the two solutions were mixed and incubated for another 20 min, and then the mixture was gently added to the cells with 1.50 mL of DMEM. The cells were maintained for 12 h, and the medium was replaced with normal DMEM.

### 2.14 Dual-luciferase reporter assay

A dual-luciferase reporter assay was performed as described in our previous study (Hu et al., 2017; Luo et al., 2016). Briefly, after being transfected with pBind FXR LBD and pGL5 luc and treated with CDCA and/or GUDCA, HEK-293T cells were lysed with 50 μL 1× passive lysis buffer. Then LAR II and Stop/Glo reagent were added respectively according to the manufacturer’s instructions. Luciferase fluorescence values were read using a multimode reader (Thermo, United States), and relative activity was normalized to Renilla luciferase fluorescence.

### 2.15 SPR assay

His-FXR LBD proteins (30–50 μg) or its mutants were coupled to CM5 chip, and different doses (2, 5, 10, 20, and 50 μM) of GUDCA were injected into the flow cells of the samples. The chip was submitted to a Biacore T200 system (GE Healthcare, United States), and the curve of binding-dissociation between GUDCA and FXR LBD was drawn.

### 2.16 Co-immunoprecipitation

Co-immunoprecipitation was performed as previously described (Hu et al., 2017). HepG2 cells transfected with flag-FXR/myc-RXRα plasmids and treated with GUDCA in a 100 mm culture plate were lysed with a gentle lysis buffer (500 μL) on ice for 30 min. The lysate was divided into two parts:50 μL for the input and 450 μL for IP tests. The input solution was boiled for denaturation, while IP solution was added with 1 μg anti-flag antibody, incubated at 4°C for 2 h, and then precipitated with protein A/G beads. The precipitated beads were washed and boiled for denaturation. Details of the procedure are described in Section 2.5.

### 2.17 Immunofluorescence

Immunofluorescence analysis was performed as previously described (Hu et al., 2017). In brief, seeded HepG2 cells transfected with flag-FXR/myc-RXRα plasmids and treated with GUDCA were permeabilized with 0.1% Triton X-100, blocked with 1% BSA and incubated with primary antibodies against FLAG and myc followed by secondary antibodies of anti-goat IgG conjugated with Cy3 and Cy5. The cells were then stained with DAPI and imaged using a laser scanning confocal microscope (Carl Zeiss, Germany). All primary antibodies were diluted at a ratio of 1:50, and the secondary antibody was diluted at 1:200.

### 2.18 Statistical analysis

Experimental data are shown as the mean ± SEM using Prism GraphPad version 5.0. A two-tailed unpaired Student’s t-test and one-way ANOVA with Dunnett’s Multiple Comparison Test was used to analyze the differences between groups in the present study. The gray value of the bands obtained by western blotting was analyzed using ImageJ version 2.3. *p*-values <0.05 were considered statistically significant at *p* < 0.01 as highly significant and *p* < 0.001 as extremely significant.

## 3 Results

### 3.1 Celastrol alleviates HCC in an orthotopic HCC rat model

**FIGURE 1:**
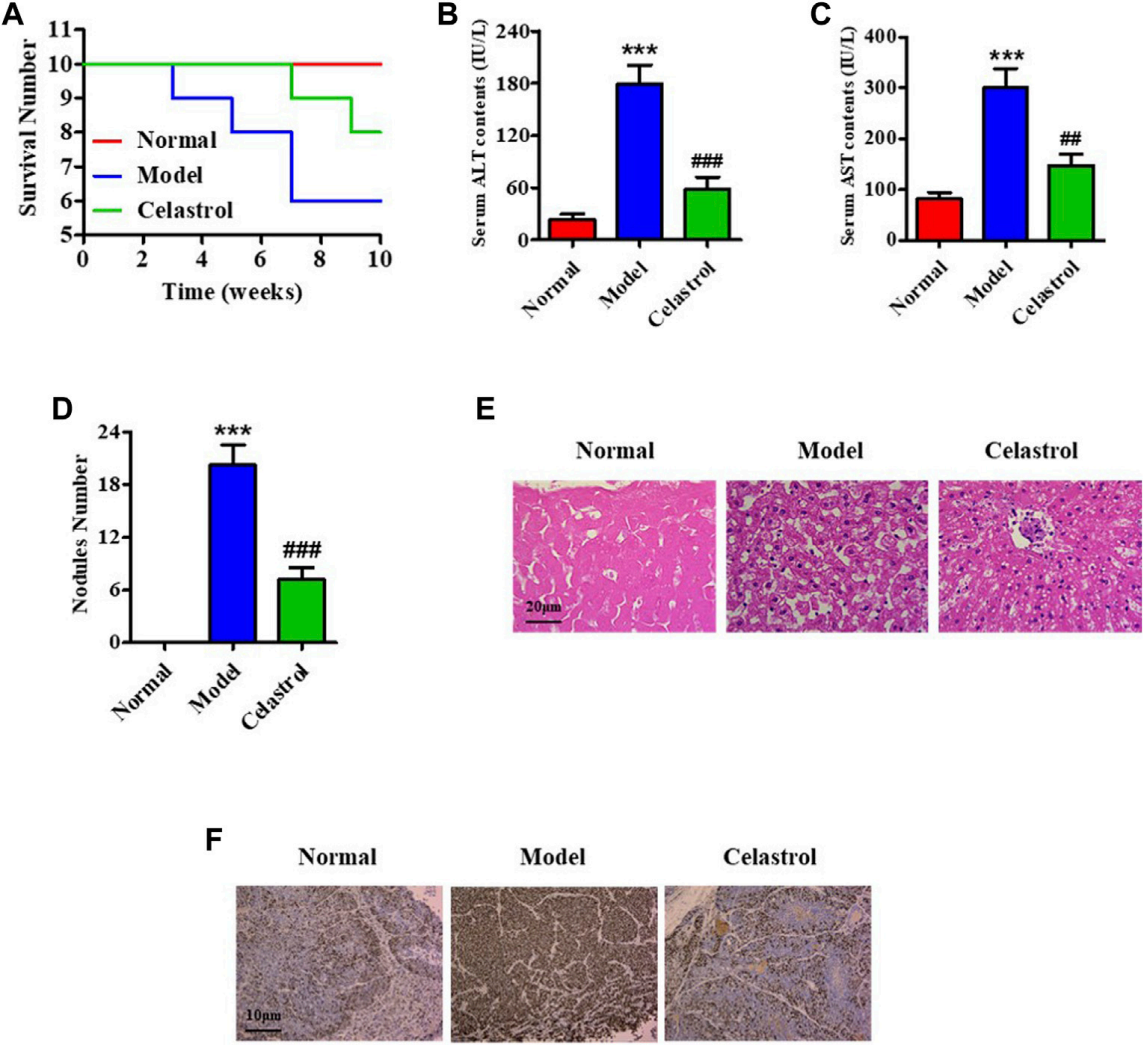
Celastrol alleviates HCC in an orthotopic rat model. **(A)** The survival number of rats. Serum levels of **(B)** ALT and **(C)** AST. **(D)** Nodule numbers of liver tissue, **(E)** H&E staining for pathology analysis. **(F)** IHC for probing ki67 expression. ***p < 0.001 vs. normal group; ^##^p < 0.01, ^###^p < 0.001 vs. model group.

We constructed a rat model with orthotopic HCC by treating the rats with DEN and administrated the model rats with celastrol or normal saline in gavage for 10 weeks. The survival curve analysis revealed a decreased survival in the model group than in the celastrol (Figure 1A). The death of rats in the model group occurred on the third week of administration (n = 1), which consistently increased until the end of the 10th week and reached 4. In contrast, in the celastrol group, the decrease in survival started in the eighth week (n = 1) which increased at the end of the 10th week (n = 2). During treatment, the body weight of rats in the model group was reduced compared to that in the normal group, and celastrol did not relieve the reduction (Supplementary Figure S1A, B). Biochemical analyses of the blood samples at the end of the 10th week of administration showed that celastrol suppressed the DEN-induced increase in serum ALT and AST levels (Figures 1B,C). The number of nodules in the liver tissue was also suppressed in the celastrol group (7.2) compared to that in the model group (20.2) (Figure 1D). Pathology images showed that the liver tissue in the model group displayed inflammatory infiltration, which was reduced in the celastrol group (Figure 1E). Furthermore, the DEN-induced increased expression of ki67, a classical proliferation marker indicating carcinoma, was suppressed by celastrol (Figure 1F). Western blot analysis revealed higher mTOR phosphorylation in the liver tissue of rats in the model group than that in the normal group, wherein this increase in mTOR phosphorylation was suppressed in the celastrol group (Supplementary Figure S1C).

### 3.2 Celastrol regulates gut microbiota and bile acid metabolism in rats with HCC

**FIGURE 2:**
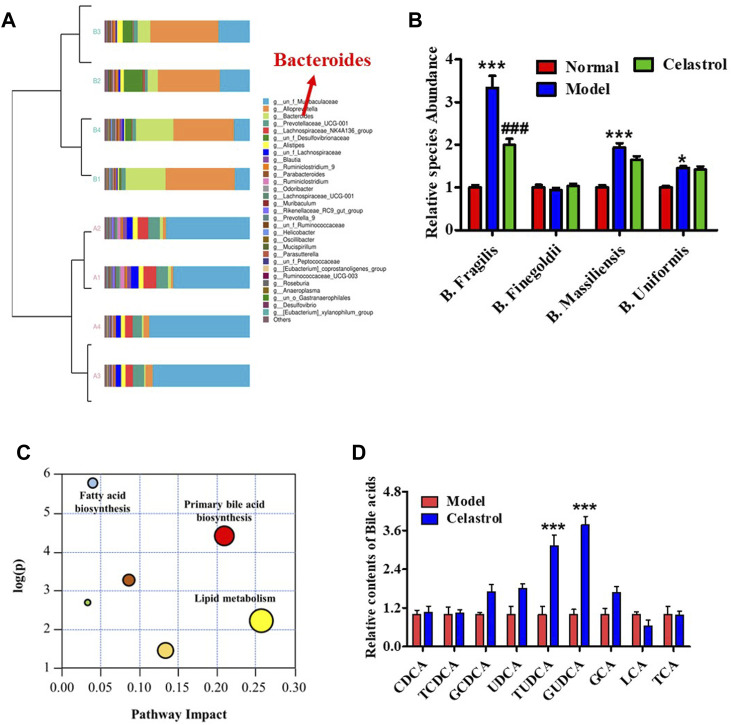
Celastrol regulates gut microbiota and bile acid metabolism in rats with HCC. **(A)** Cluster diagram of bacterial structure. **(B)** Different species abundance of *Bacteroides*, *p < 0.05, ***p < 0.001 vs. normal group; ^###^ *p* < 0.001 vs. model group. **(C)** Metabolism pathway analysis. **(D)** Bile acids level in liver, ***p < 0.001 vs. model group.

The rats administered celastrol had loose bowels. To evaluate the effect of celastrol on gut microbiota, we performed 16S rDNA sequencing and qPCR experiments. A Venn diagram of OTU distribution showed 273 overlapping OTUs, 69 individual OTUs in the gut microbiota of the normal group, and 26 individual OTUs in the model group (Supplementary Figure S2A). The PCA scatter plot revealed significant differences between the gut microbiota composition of the normal and model groups (Supplementary Figure S2B). Analysis of the bacterial community structure of fecal samples from the normal and model groups also revealed differences (Supplementary Figure S2C and Supplementary Figures S3A, S3B). Cluster analysis of bacterial community structure showed that the abundance of different *Bacteroides* species was highly different in the gut of the normal and model group rats (Figure 2A). To further identify the different strains, we detected the richness of four sub-strains of *Bacteroides*, *Bacteroides fragilis*, *Bacteroides finegoldii, Bacteroides massiliensis and Bacteroides uniformis*. As shown in Figure 2B, DEN increased the richness of *B. Fragilis, B. Massiliensis and B. Uniformis*. However, celastrol significantly suppressed the DEN-induced increase in *B. fragilis* richness, demonstrating that *B. fragilis* could be essential for celastrol-mediated alleviation of HCC (Figure 2B). Subsequent analysis of the *B. fragilis*-associated metabolism pathway showed that primary bile acid biosynthesis was one of the most promising pathways for HCC progression (Figure 2C). In addition, data from several liver bile acids showed that celastrol increased the levels of GCDCA, UDCA, TUDCA, and GUDCA, while the increase in GUDCA was the most evident (Figure 2D). The structure of GUDCA is shown in Supplementary Figure S3C.

### 3.3 GUDCA suppresses the cellular proliferation in HepG2 cells

**FIGURE 3:**
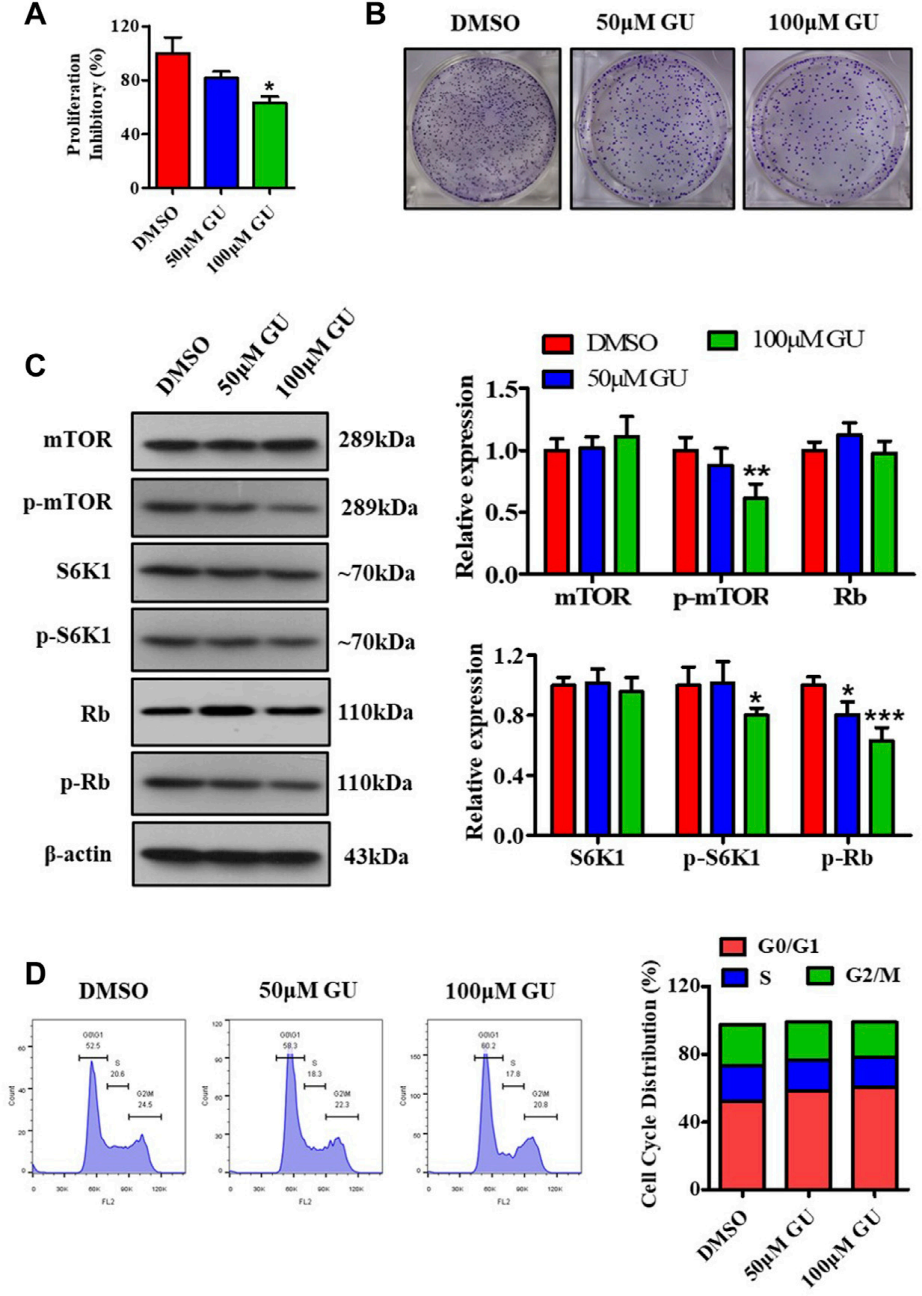
GUDCA suppresses the cellular proliferation in HepG2 cells. **(A)** MTT assay for cellular proliferation. **(B)** Clone formation experiment to assess the clone number. **(C)** Western blot for probing the expression of mTOR, p-mTOR, S6K1, p-S6K1, and p-Rb. **(D)** Flow cytometry for detecting cell cycle distribution. *p < 0.05 and **p < 0.01 vs. DMSO group. GU means Glycoursodeoxycholic acid (GUDCA).

To evaluate the effect of GUDCA on the proliferation of hepatoma carcinoma cells, MTT assay, clone formation test, western blotting assay for probing the mTOR/S6K1 pathway, and flow cytometry assay for detecting cell cycle distribution were performed in HepG2 cells. The MTT assay showed that 50 and 100 μM GUDCA inhibited the proliferation of HepG2 cells in a dose-dependent manner, and the proliferation inhibition ratio at 100 μM was statistically different (Figure 3A). The clone formation test showed a similar effect of GUDCA with the MTT assay, in which GUDCA inhibited the cellular clone number in a dose-dependent manner in HepG2 cells (Figure 3B). Western blot analysis showed that GUDCA suppressed the phosphorylation of mTOR, S6K1, and Rb (Figure 3C), demonstrating that GUDCA regulates the mTOR/S6K1/Rb pathway, which regulates the cell cycle distribution and affects cellular proliferation. Furthermore, flow cytometry data revealed that GUDCA arrested the G1 phase of the cell cycle in HepG2 cells (Figure 3D), wherein expression of G0/G1 phase-related proteins, including cyclinD1, CDK4, CDK6, and cdc25A was detected using western blotting, indicating that these proteins are also regulated by GUDCA (Supplementary Figure S5).

### 3.4 GUDCA is identified as an antagonist of FXR

**FIGURE 4:**
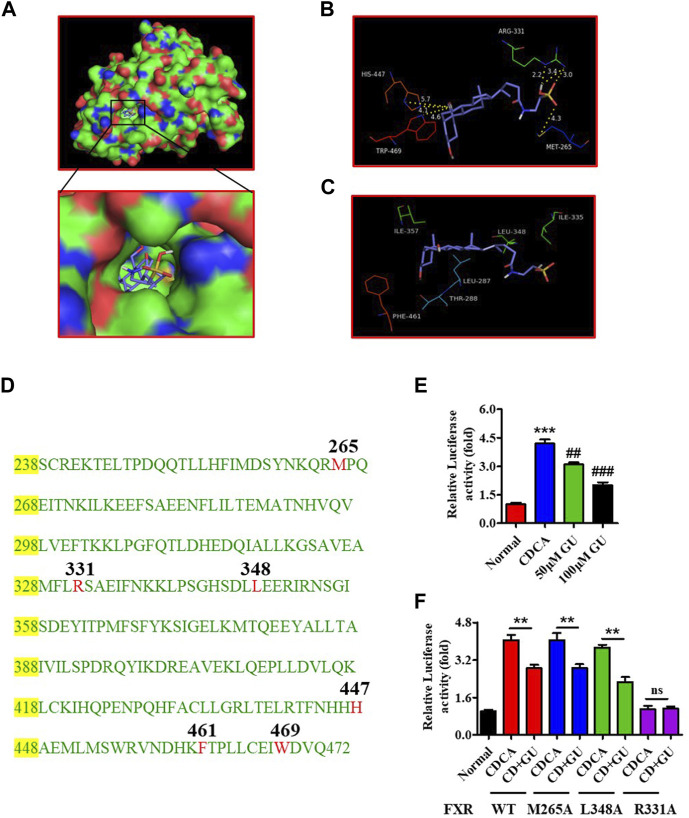
GUDCA is an antagonist of FXR. **(A)** The docked conformation of GUDCA with FXR (3DCT); **(B)** H-bond interaction of GUDCA with FXR residues; **(C)** Hydrophobic interaction of GUDCA with FXR residues; **(D)** Amino acid sequence of FXR LBD; **(E)** Reporter assay for detecting the effect of GUDCA on the transcriptional activity of FXR, ***p < 0.001 vs. normal group; ^##^ *p* < 0.01, ^###^ *p* < 0.001 vs. CDCA group; **(F)** Reporter assay for detecting the effect of GUDCA on the transcriptional activity of FXR mutant, **p < 0.01 vs. CDCA group; ns, not significant. GU means Glycoursodeoxycholic acid (GUDCA).

Bile acid is an important bioactive metabolite that plays a crucial role in the physiological and pathological processes by interacting with nuclear receptors containing FXR. Molecular simulations and reporter assays were performed to evaluate the interaction between GUDCA and FXR. Molecular docking images showed that GUDCA bonded to FXR in the previously reported cave (3DCT), which was the location of GW4064 binding (Figure 4A; Supplementary Figure S6A), a classic FXR ligand, and displayed an extremely similar binding conformation to GW4064 (Supplementary Figure S6B). The lowest binding energy of GUDCA with FXR reached −11.59 kJ/mol (Supplementary Figures S6C–E), revealing the well-binding potential of GUDCA with FXR. The key binding residue analysis showed that FXR M265, R331, H447, and W469 residues had H-bond interactions with GUDCA (Figure 4B), and FXR L348 and F461 residues had hydrophobic interactions (Figure 4C). Figure 4D shows the amino acid sequence of FXR LBD. Furthermore, the interaction between GUDCA and FXR was confirmed using SPR and reporter assays. The binding-dissociation curve obtained by SPR revealed that GUDCA binds to the FXR LBD in a dose-dependent manner (Supplementary Figure S7). A reporter assay revealed that GUDCA suppressed the CDCA-induced transcriptional activity of FXR (Figure 4E). Furthermore, FXR mutant data showed that FXR R331 affected the effect of GUDCA on FXR transcriptional activity (Figure 4F).

### 3.5 GUDCA affects FXR interaction with RXRα in HepG2 cells

**FIGURE 5:**
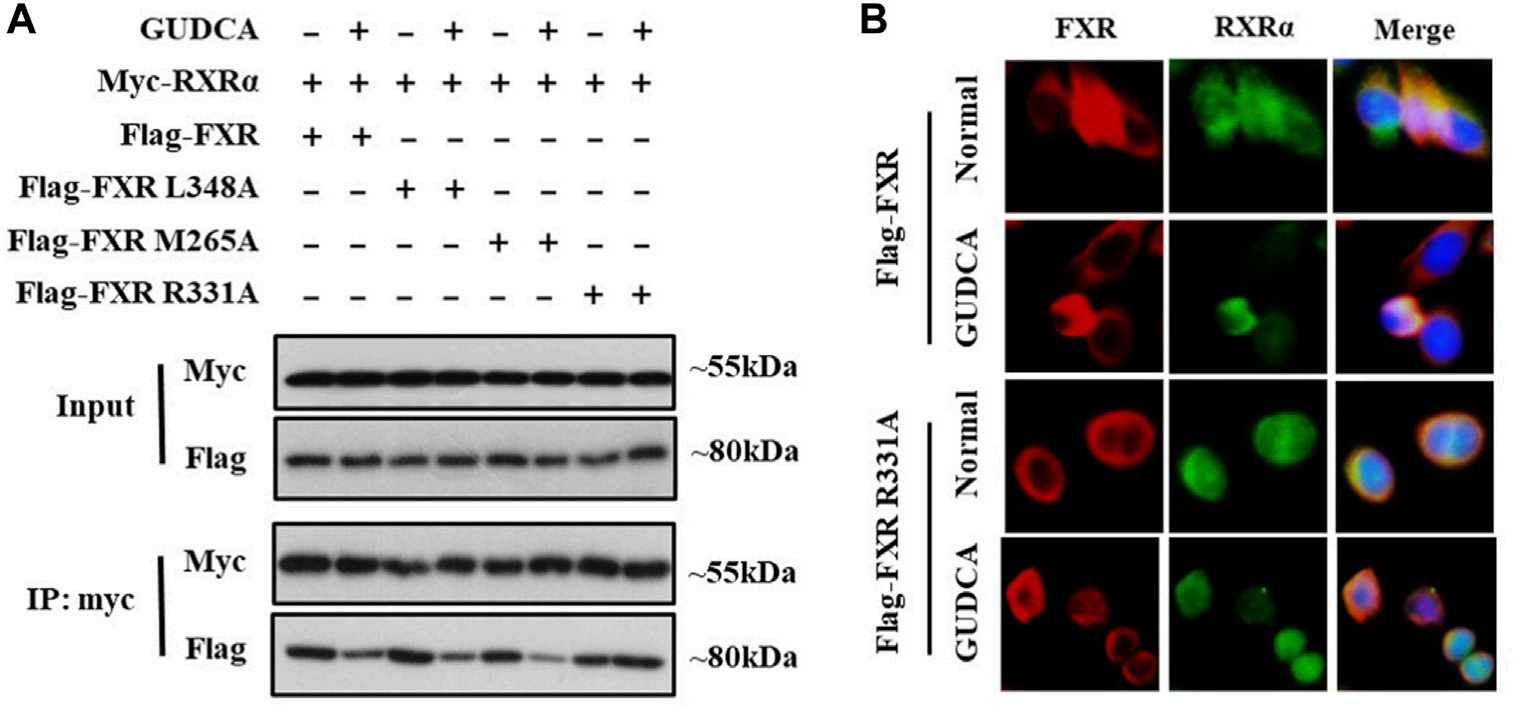
GUDCA affects FXR interaction with RXRα in HepG2 cells. **(A)** Co-IP for detecting the interaction of FXR with RXRα; **(B)** Immunofluorescence assay for detecting the colocation of FXR with RXRα.

RXRα is an important nuclear receptor that participates in several bio-behaviors, including proliferation *via* its interaction with itself (forming homodimers) or with many other nuclear receptors such as FXR (forming heterodimers). To confirm whether GUDCA affects the interaction of FXR with RXRα, we performed co-immunoprecipitation and immunofluorescence analyses in HepG2 cells. Co-immunoprecipitation data showed that GUDCA suppressed the interaction of FXR with RXRα in the transfection conditions of FLAG-FXR, FLAG-FXR L348A, and FLAG-FXR M265; however, the suppressive effect of GUDCA on the interaction of FXR with RXRα weakened in the transfection conditions of flag-FXR R331A (Figure 5A). Immunofluorescence analysis revealed that GUDCA significantly inhibited the colocation of transiently transfected FXR and RXRα, wherein the inhibitory effect of GUDCA on the colocation of transiently transfected FXR R331A and RXRα was slight (Figure 5B).

### 3.6 FXR is essential for GUDCA-mediated mTOR pathway-associated proliferation in HepG2 cells

**FIGURE 6:**
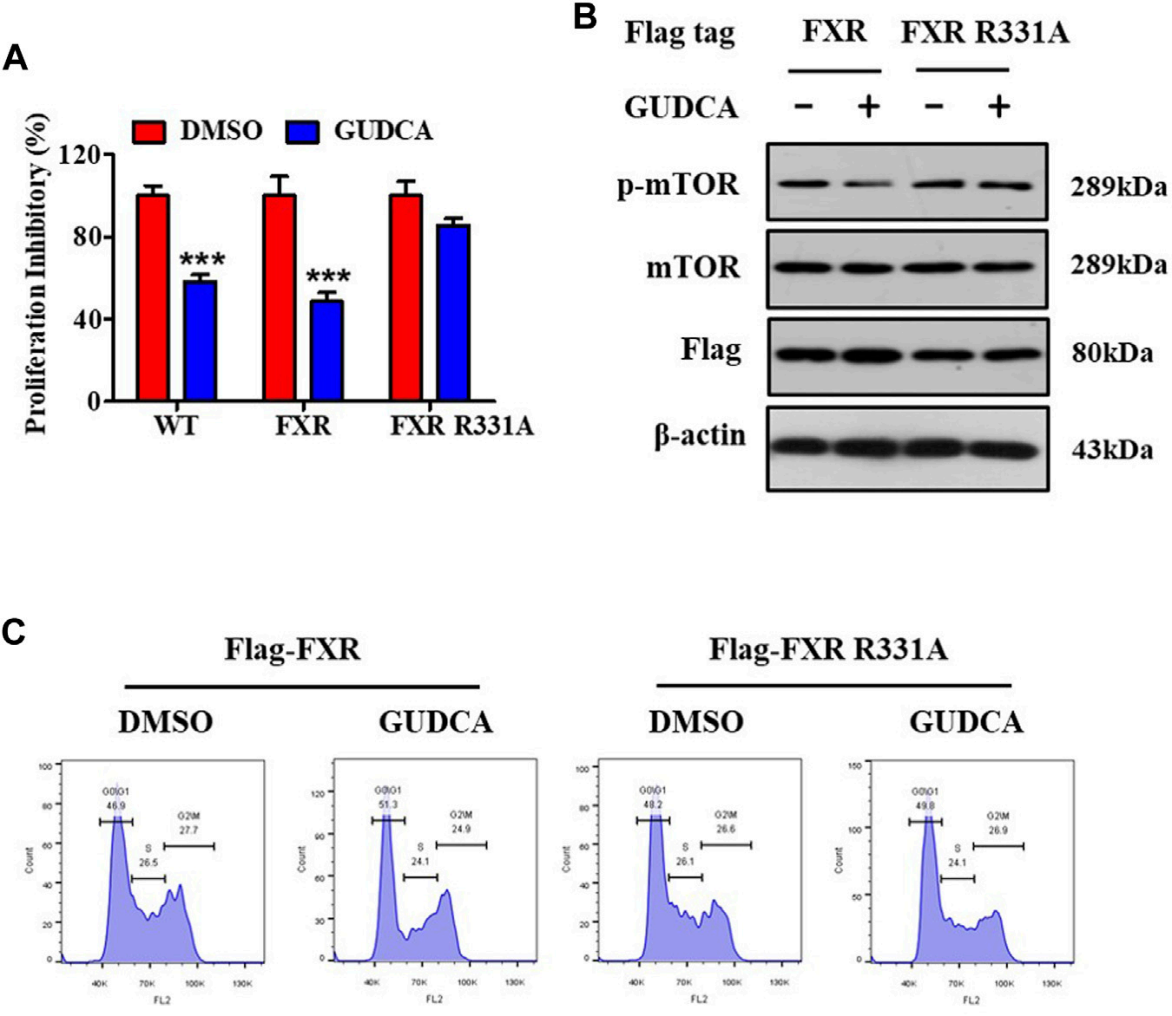
FXR is essential for GUDCA-alleviating mTOR pathway-associated proliferation in HepG2 cells. **(A)** MTT assay for detecting cellular proliferation, ***p < 0.001 compared to DMSO group; **(B)** Western blot for probing the expressions of p-mTOR, mTOR, and Flag; **(C)** Flow cytometry for detecting cell cycle distribution.

To further elucidate the mechanism of GUDCA-alleviating hepatoma carcinoma cell proliferation and clarify the role of FXR in the effect of GUDCA, the plasmids of wild-type and mutant FXR were transfected into HepG2 cells, and the effect of GUDCA on proliferation was evaluated using MTT assay, western blotting, and flow cytometry. Proliferation inhibitory data demonstrated a proliferation inhibitory effect in HepG2 cells subjected to transient transfection of FXR siRNA/flag-FXR in response to GUDCA, which was similar to that in wild-type HepG2 cells; however, HepG2 cells subjected to transient transfection with FXR siRNA/flag-FXR R331A lost their proliferation inhibitory effect (Figure 6A). Consistently, mTOR phosphorylation was inhibited, and cell cycle G0/G1 phase was arrested in HepG2 cells subjected to FXR siRNA/flag-FXR transfection in response to GUDCA; however, contrasting results were observed in HepG2 cells subjected to transient transfection of FXR siRNA/flag-FXR R331A (Figures 6B,C and Supplementary Figure S8).

### 3.7 GUDCA improves the alleviating effect of celastrol on HCC in a rat model

**FIGURE 7:**
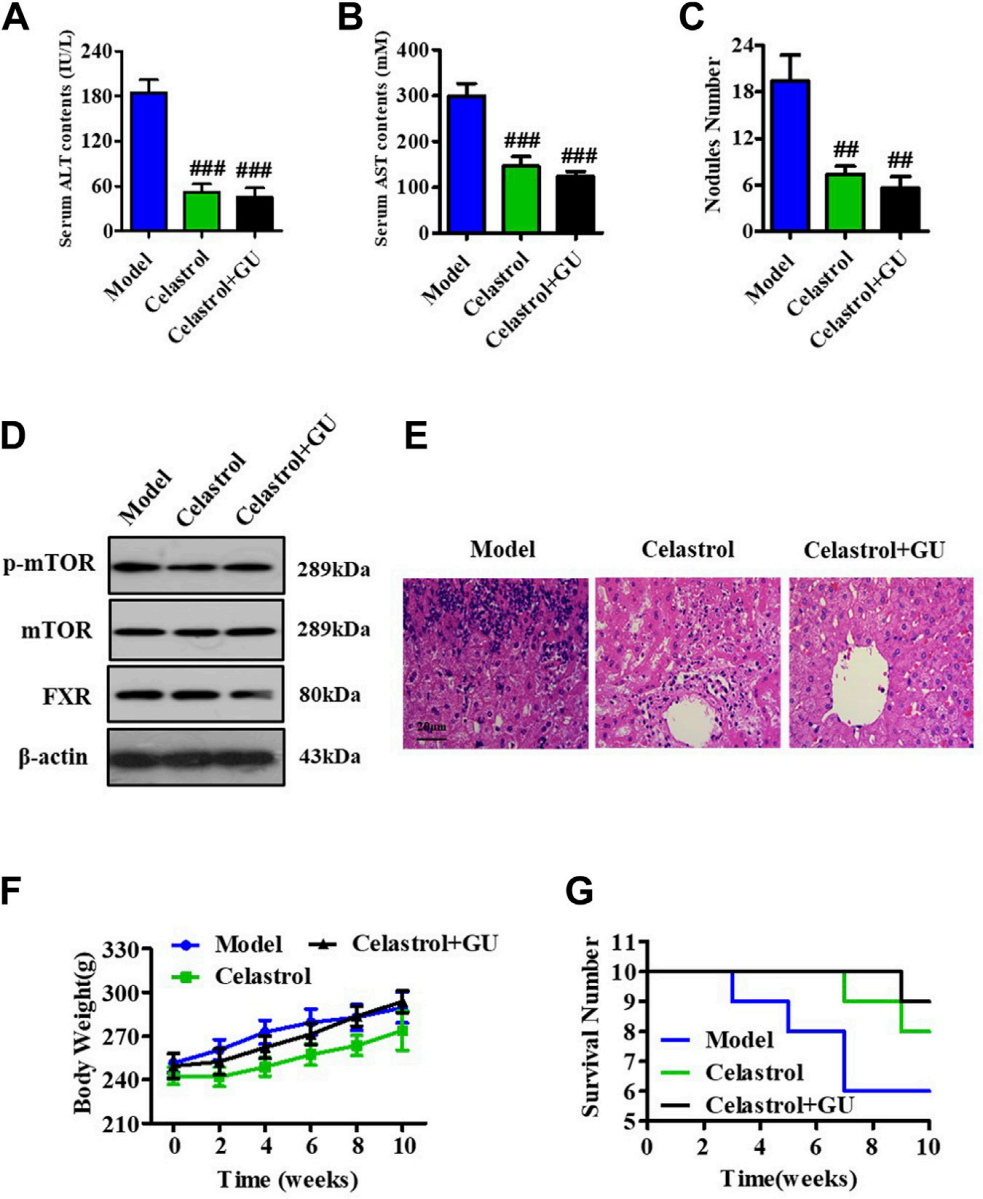
GUDCA improves the alleviating effect of celastrol on HCC in an orthotopic rat model. Serum levels of **(A)** ALT and **(B)** AST; **(C)** Nodule numbers of liver tissue; **(D)** Western blot for probing the expressions of p-mTOR, mTOR, and FXR; **(E)** H&E staining for pathology analysis; **(F)** The curve of body weight; **(G)** Survival number of rats. ^##^ *p* < 0.01 and ^###^ *p* < 0.001 compared to model group. GU means Glycoursodeoxycholic acid (GUDCA).

To explore the role of GUDCA in the celastrol-mediated alleviation of HCC, we constructed a rat model of HCC using DEN and evaluated the combined effect of celastrol and GUDCA. The rats treated with the combination of celastrol and GUDCA displayed significant inhibitory effects on serum levels of ALT and AST, liver nodule number, mTOR phosphorylation, and inflammation infiltration, which was similar to the effect of celastrol alone (Figures 7A–E). Moreover, the combination of celastrol and GUDCA alleviated body weight loss to a certain extent compared to celastrol alone during treatment (Figure 7F). The combination of celastrol and GUDCA improved the survival rate of rats; only one death was recorded at the end of the 10th week in the combined treatment group, wherein three deaths (one at the eighth week and two at the end of the 10th week) by the end of the 10th week were recorded in the only celastrol treatment group (Figure 7G).

## 4 Discussion

For decades, celastrol has been used in folk medicine and clinical practice to treat cancer, including HCC. Because of its broad beneficial biological effects, such as inflammation suppression, cellular proliferation inhibition (Fang and Chang, 2021; Chen et al., 2022), the lipid absorption alleviation (Zhang et al., 2019; Hu et al., 2021), celastrol has garnered the research focuses of natural medicinal agents. Studies unveiling the mechanism of the hepato-protective effect of celastrol have demonstrated that its functions are mainly regulated *via* caspase-dependent apoptosis (Shen et al., 2021) and mTOR/AKT-associated proliferation (Li et al., 2018). Our previous study revealed that celastrol directly binds to the Nur77 nuclear receptor, clears inflamed mitochondria, and alleviates liver injury (Hu et al., 2017). All these mechanisms of action of celastrol on hepatic diseases focus on its direct effect on the signaling process in hepatocytes and liver tissues. Furthermore, the gut microbiota has been reported to play an important role in liver function and hepatic diseases. The present study demonstrates that celastrol regulates the bacterial community structure of the gut and suppresses the abundance of *B. fragilis*, which may have an important role in celastrol-mediated HCC alleviation. This result extends the direct mechanism of HCC alleviating effects of celastrol and provides a new direction to explore the mechanism of its other functions.

*Bacteroides fragilis* is an important member of *Bacteroides* and plays an essential role in the pathological progression of cancers. It has also been shown that *B. fragilis* could cause intestinal inflammation and tissue injury, eventually leading to colorectal cancer (Cheng et al., 2020; Haghi et al., 2019). In addition, *B. fragilis* has also been shown to be associated with gastric carcinoma, lung abscess, renal diseases, *etc.* In this study, we found that celastrol that alleviated HCC suppressed the abundance of *B. fragilis* in rats. Several studies have revealed that *B. fragilis* participates in liver bile acid metabolism and regulates hepatic function. Concordantly, the present study showed that celastrol is involved in maintaining the levels of liver bile acids; it significantly increased the level of GUDCA while suppressing the abundance of *B. fragilis*. These results imply that *B. fragilis*–GUDCA may be one of the mechanisms underlying celastrol-mediated alleviation of HCC. However, the present study could not explain the inter-relationships of *B. fragilis*, bile acids metabolism, and HCC alleviating effects of celastrol; therefore, further studies are required to gain further insights. In the future, we propose to evaluate the detailed relationship between therapeutic effects of celastrol against HCC and the alterations in bile acid levels and its upstream pathways *via* employing *B. fragilis* to treat rat with HCC.

Bile acids is an important component of bile, mainly in the enterohepatic circulation system. In response to the gut microbiota, the contents and types of bile acids are changed in the intestine, absorbed into the liver *via* enterohepatic circulation, and exert a regulatory effect on hepato-metabolism. Bile acid metabolism plays an important role in the protection of hepatotoxicity. FXR, a member of the nuclear receptor family, is highly expressed in the liver and intestinal tissues to maintain metabolic homeostasis. Several studies have shown that FXR interacts with bile acids such as CDCA, regulates the heterodimerization of FXR with other nuclear receptors, and exerts its multifunction, including lipid metabolism (Zhang et al., 2019; Ramírez-Pérez et al., 2017), hepato-inflammation (Han et al., 2018), and cellular proliferation. This study revealed that the bile acid GUDCA could bind to FXR and suppress its transcriptional activity. Furthermore, FXR was confirmed to be essential for the effects of GUDCA on the mTOR-associated cell cycle G0/G1 arrest and cellular proliferation. These findings enrich the understanding of the function of bile acids and the mechanisms regulating FXR.

RXRα is a member of the nuclear receptor family; RXRα interacts with approximately 1/3 of the nuclear receptor family members to form a heterodimer or homodimer and acts in many physiological and pathological processes. Numerous studies have confirmed that several antitumor agents can target RXRα to regulate cell cycle distribution and inhibit the proliferation of tumor cells (Zhang et al., 2016). However, it is rarely reported that the heterodimer of RXRα/FXR in response to medicinal agents participates in the progression of HCC. In the present study, we verified that GUDCA inhibits the interaction of RXRα with FXR during the suppression of HCC proliferation in HepG2 cells and that the FXR R331 mutant weakened the effect of GUDCA on the interaction of RXRα with FXR and cellular proliferation. In conclusion, the findings of this study demonstrate that the RXRα/FXR interaction is essential for GUDCA-mediated suppression of hepatocarcinoma cell proliferation. Combining our previous research that celastrol could directly bind to the Nur77 nuclear receptor to clear up inflamed mitochondria and repair liver injury, the present study elucidated that the effects of celastrol are regulated *via* a mechanism of synergistic functions *via* directly and/or indirectly targeting three nuclear receptors, FXR, RXRα, and Nur77, which provides a novel strategy for the design of antitumor agents.

## 5 Conclusion

**FIGURE 8:**
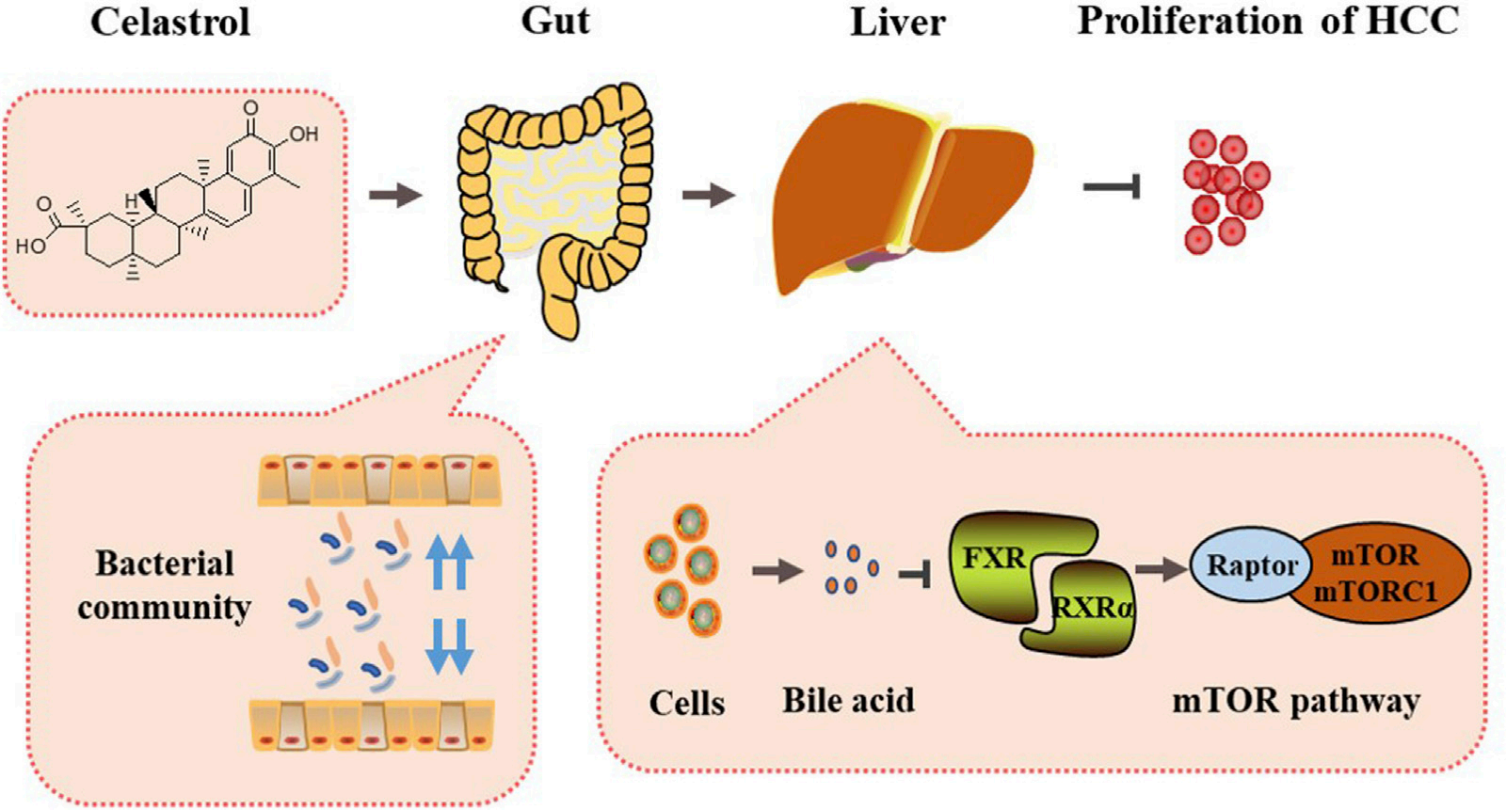
Mechanism summary of celastrol alleviating proliferation of hepatocellular carcinoma *via* regulation of the interaction of FXR with RXRα modulated by gut microbiota-associated bile acid metabolism.

The present study revealed a mechanism of celastrol-alleviating HCC that celastrol could regulate the gut microbiota and liver bile acid metabolism, inhibit the interaction of FXR with RXRα in the liver, induce mTOR/S6K1-related cell cycle G0/G1 phase arrest, and eventually alleviate the proliferation of HCC (Figure 8).

## Data Availability

The original contributions presented in the study are included in the article/Supplementary Material, further inquiries can be directed to the corresponding author.
