# Supplementary material for: Celastrol-regulated gut microbiota and bile acid metabolism alleviate hepatocellular carcinoma proliferation by regulating the interaction between FXR and RXRα in vivo and in vitro
Source: Front Pharmacol. 2023 Feb 15;14:1124240. doi: 10.3389/fphar.2023.1124240 (PMC9975715; doi:10.3389/fphar.2023.1124240)
Supplement: Supplementary file 1 [file Table1.pdf]

# Supplemental Materials

## Celastrol-regulated gut microbiota and bile acid metabolism alleviate hepatocellular carcinoma proliferation by regulating the interaction between FXR and RXRα *in vivo* and *in vitro*

Dequan Zeng [1, 2, 3], Lipen Zhang [1, 2], Qiang Luo [1, 2, *]

[1]Key Laboratory of Design and Assembly of Functional Nanostructures, Fujian Institute of Research on the Structure of Matter, Chinese Academy of Sciences, Fuzhou, China

[2]Department of Translational Medicine, Xiamen Institute of Rare Earth Materials, Chinese Academy of Sciences, Xiamen, China

[3]School of Pharmaceutical Science, Xiamen University, Xiamen, China

*Correspondence: Qiang Luo, luoq@fjirsm.ac.cn

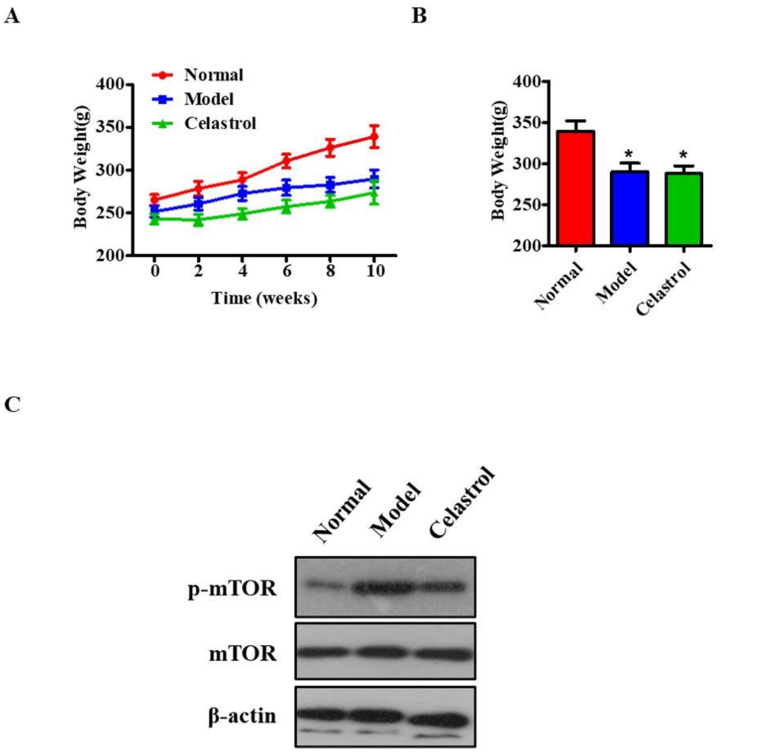


Figure S1 Celastrol alleviates HCC in rat model, related to Figure 1.

A. Body weight curve of rats during celastrol-alleviating Hepatocellular Carcinoma.

B. Body weight of rats at 14th week.

C. Celastrol suppresses mTOR pathway in liver tissue of rat Hepatocellular Carcinoma.

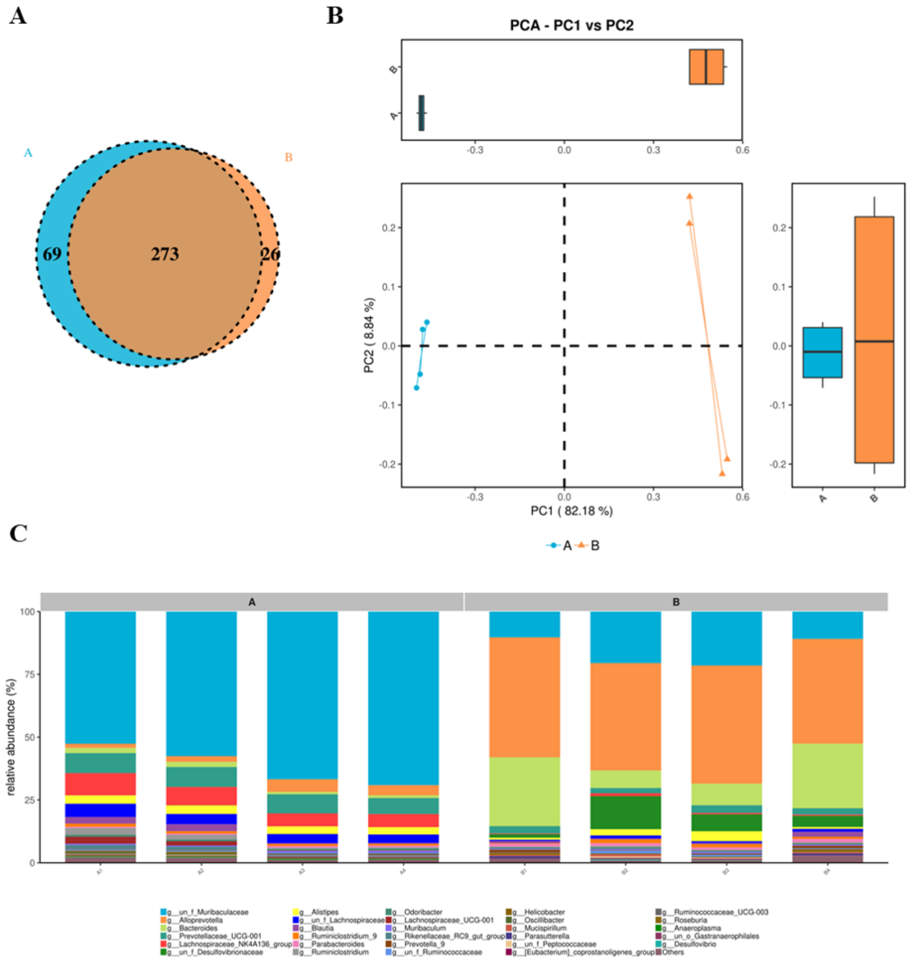


Figure S2 Celastrol regulates gut microbiota in rat with HCC, related to Figure 2.

A. Venn diagram for OUT distribution of samples.

B. PCA scatter plot analyzing the similarity of the samples between normal group and model group.

C. Analysis of bacterial community structure of fecal samples for the different bacterial between normal group and model group.

A

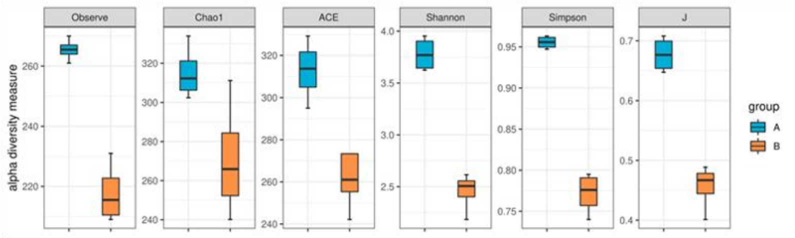


B

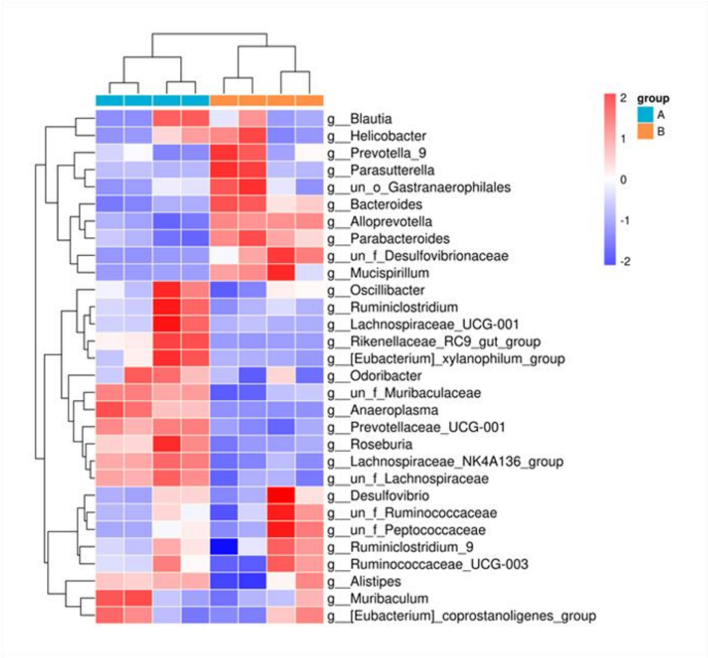


C

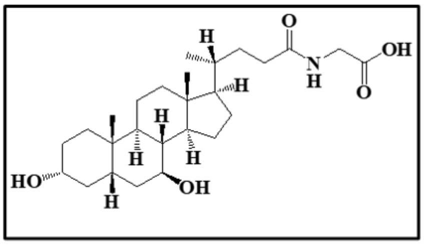

Figure S3 Celastrol regulates gut microbiota in rat with HCC, related to Figure 2.

A. α-diversity analysis reflecting the community richness (ACE, Chao1, Observed species), diversity (Shannon and Simpson) and niiformity (J).

B. Cluster heatmap analysis of samples and species reflecting the similarity of samples and community structure.

C. The structure of bile acid GUDCA.

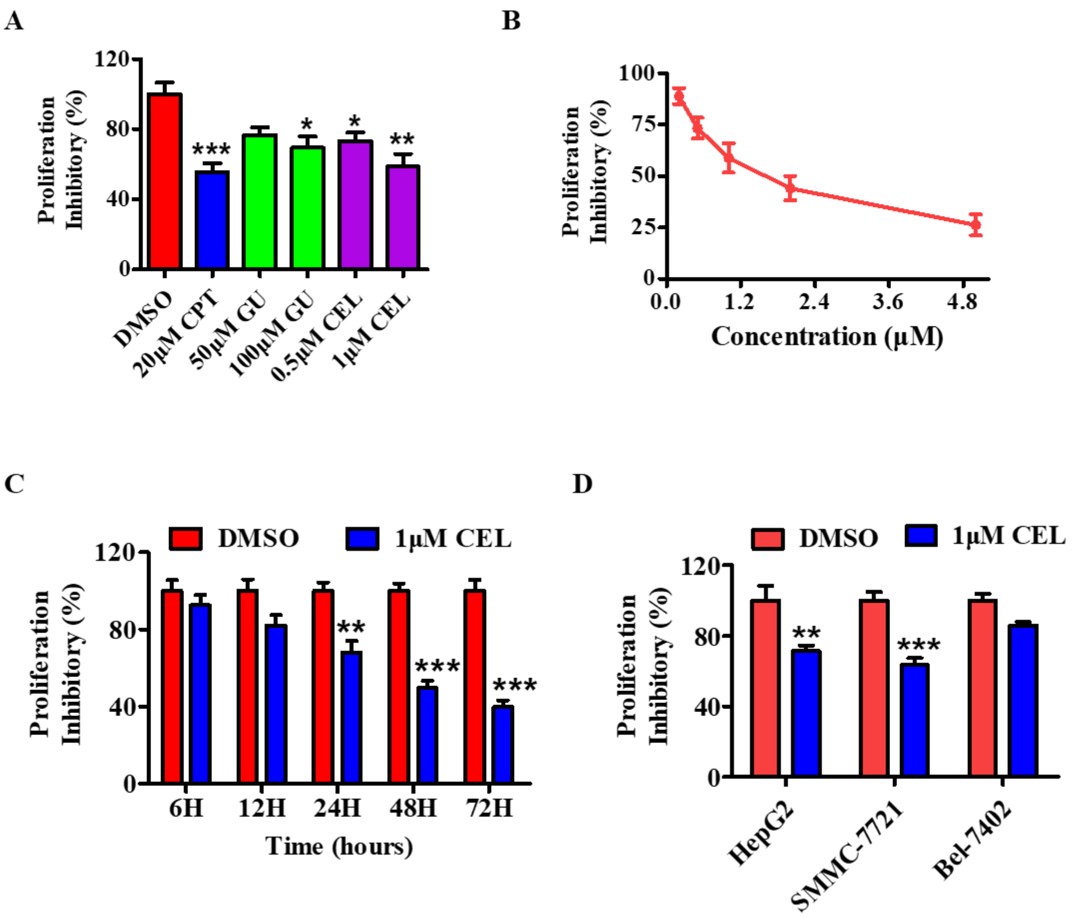


Figure S4 Celastrol inhibit the proliferation of hepato-carcinoma cells. Related to Figure 3.

(CPT for cisplatin, GU for GUDCA and CEL for celastrol)

A. Anti-proliferation effect of GUDCA and celastrol on HepG2 cells.

B. The curve of proliferation-concentration by celastrol in HepG2 cells.

C. Anti-proliferation effect of 1 μM celastrol on HepG2 cells in the time course.

D. Anti-proliferation effect of 1 μM celastrol on the indicated HCC cell lines.

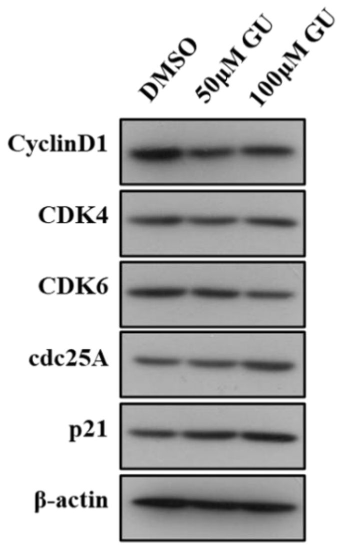


Figure S5 GUDCA regulates the expression of cell cycle G0/G1-associated proteins cyclin D1, CDK4, CDK6, cdc25A and p21. Related to Figure 3.

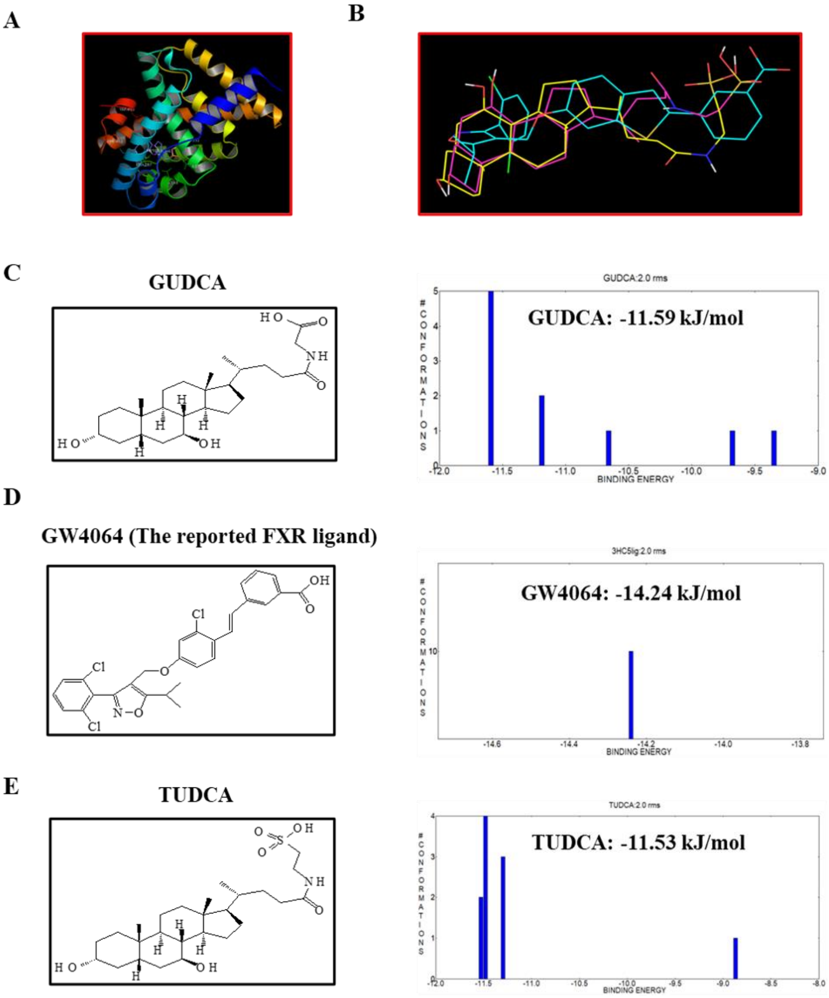


Figure S6 GUDCA could bind to FXR similar to the reported FXR ligand GW4064. Related to Figure 4.

A. The docked conformation of GUDCA with FXR (3DCT) indicated that GUDCA could dock into FXR.

B. The mapping of GUDCA, GW4064 and TUDCA showed GUDCA have the extremely similar conformation pose with the reported FXR ligand GW4064.

C-E. The structures of GUDCA (C), GW4064 (D) and TUDCA (E), and their lowest binding energy with FXR demonstrates GUDCA could bind to FXR.

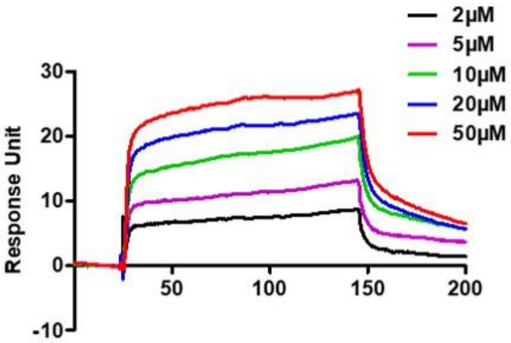


Figure S7 FXR R331 is essential to the binding of GUCDA to FXR, related to Figure 4. SPR assay showed that GUDCA could bind to FXR in dose-dependent manner.

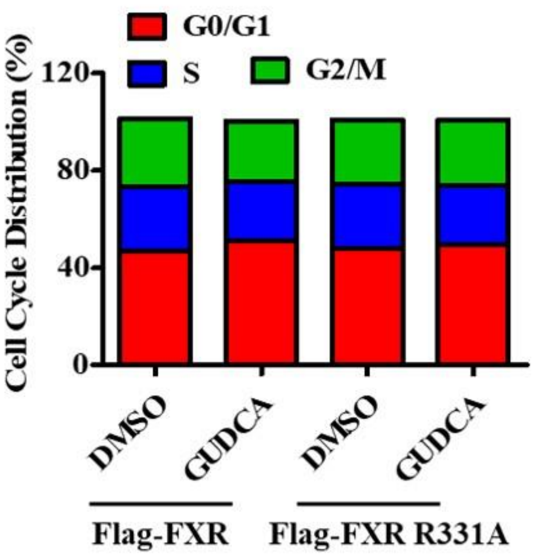


Figure S8 The statistics analysis of GUDCA-regulating cell cycle distribution, related to Figure 6.
