# Supplementary material for: Celastrol-regulated gut microbiota and bile acid metabolism alleviate hepatocellular carcinoma proliferation by regulating the interaction between FXR and RXRα in vivo and in vitro
Source: Front Pharmacol. 2023 Feb 15;14:1124240. doi: 10.3389/fphar.2023.1124240 (PMC9975715; doi:10.3389/fphar.2023.1124240)

The raw data could be available from the link: <https://www.jianguoyun.com/p/DYcHmeIQ8OWiCxiNrO0EIAA>


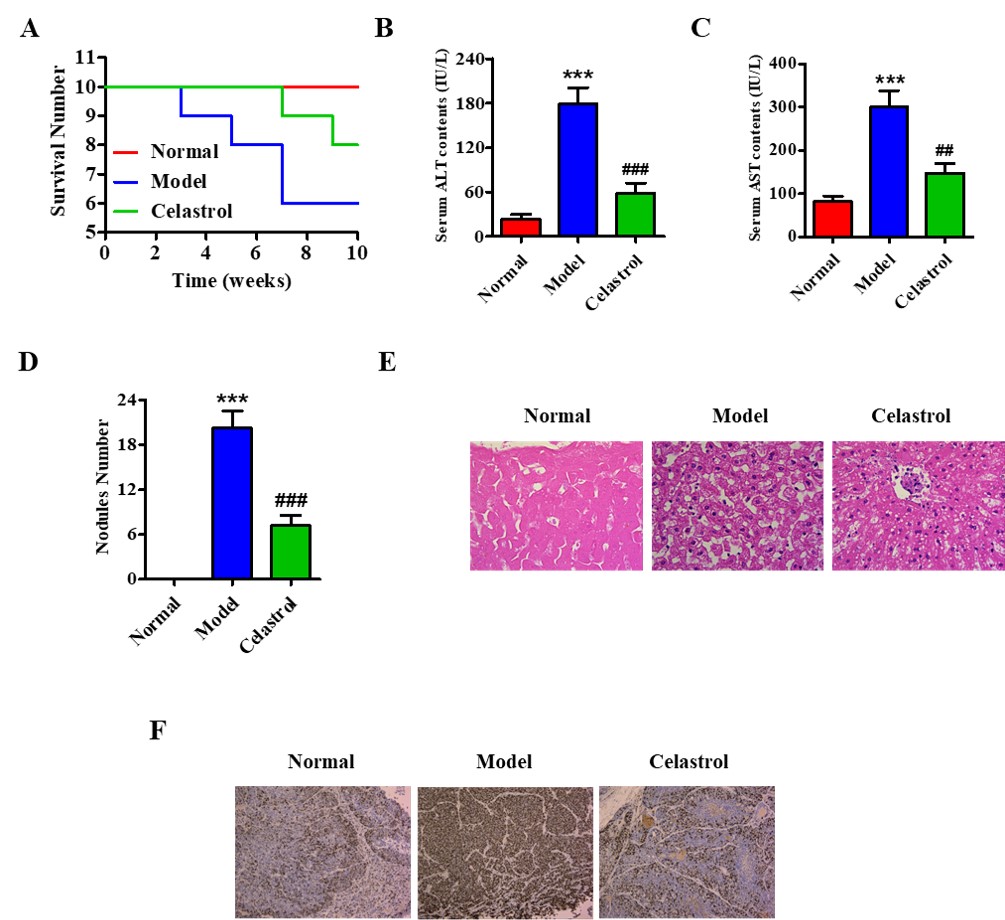

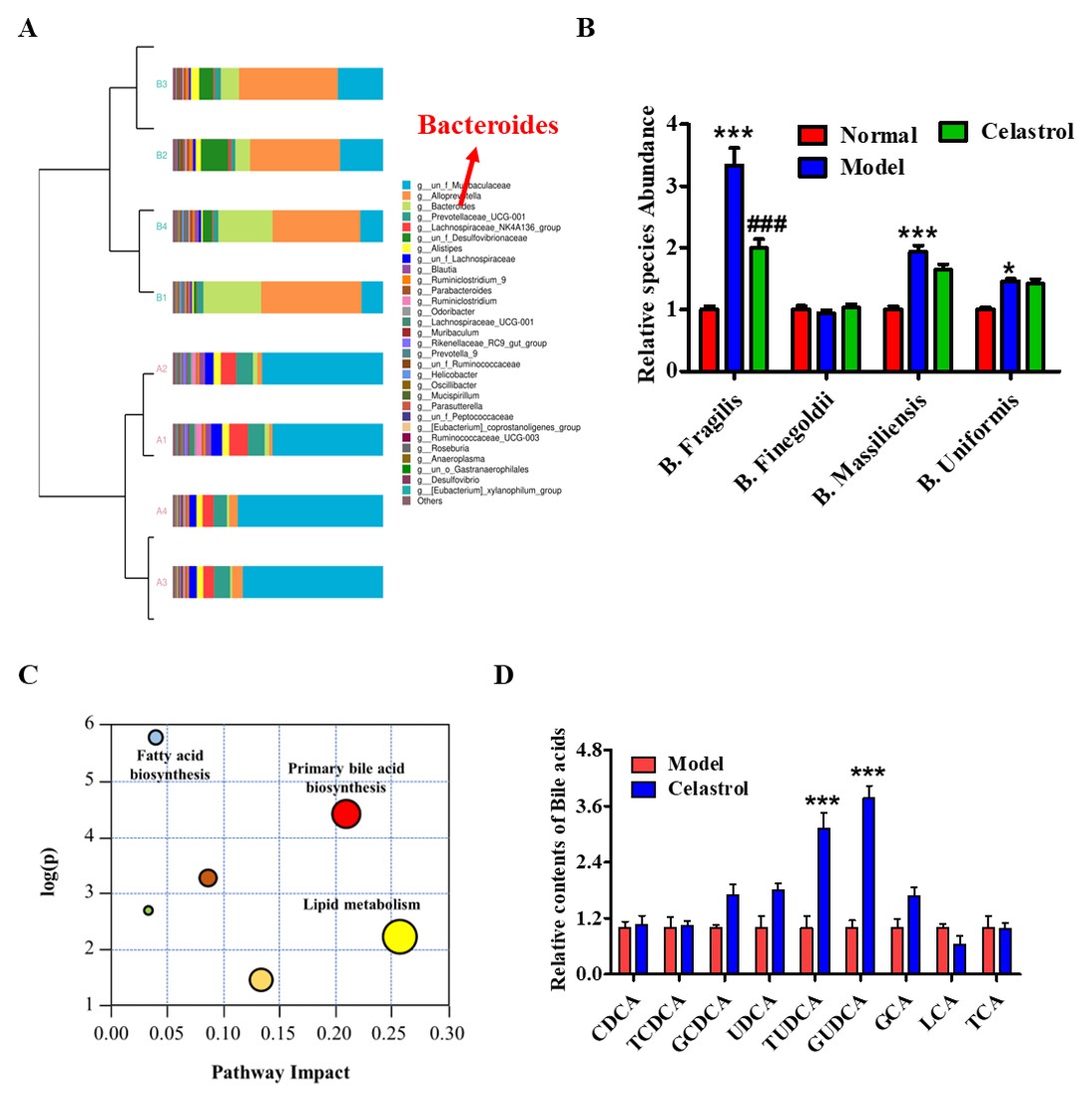

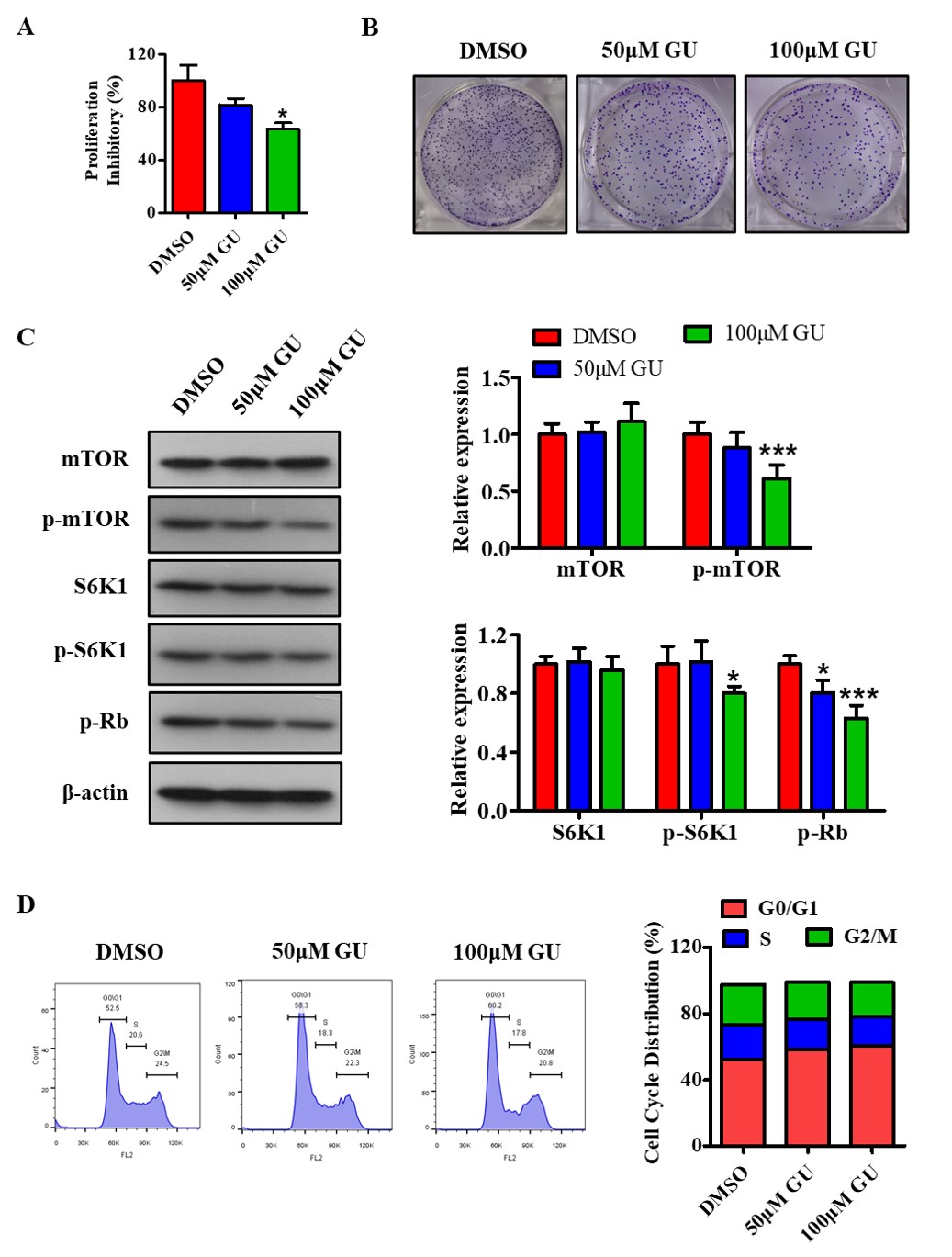

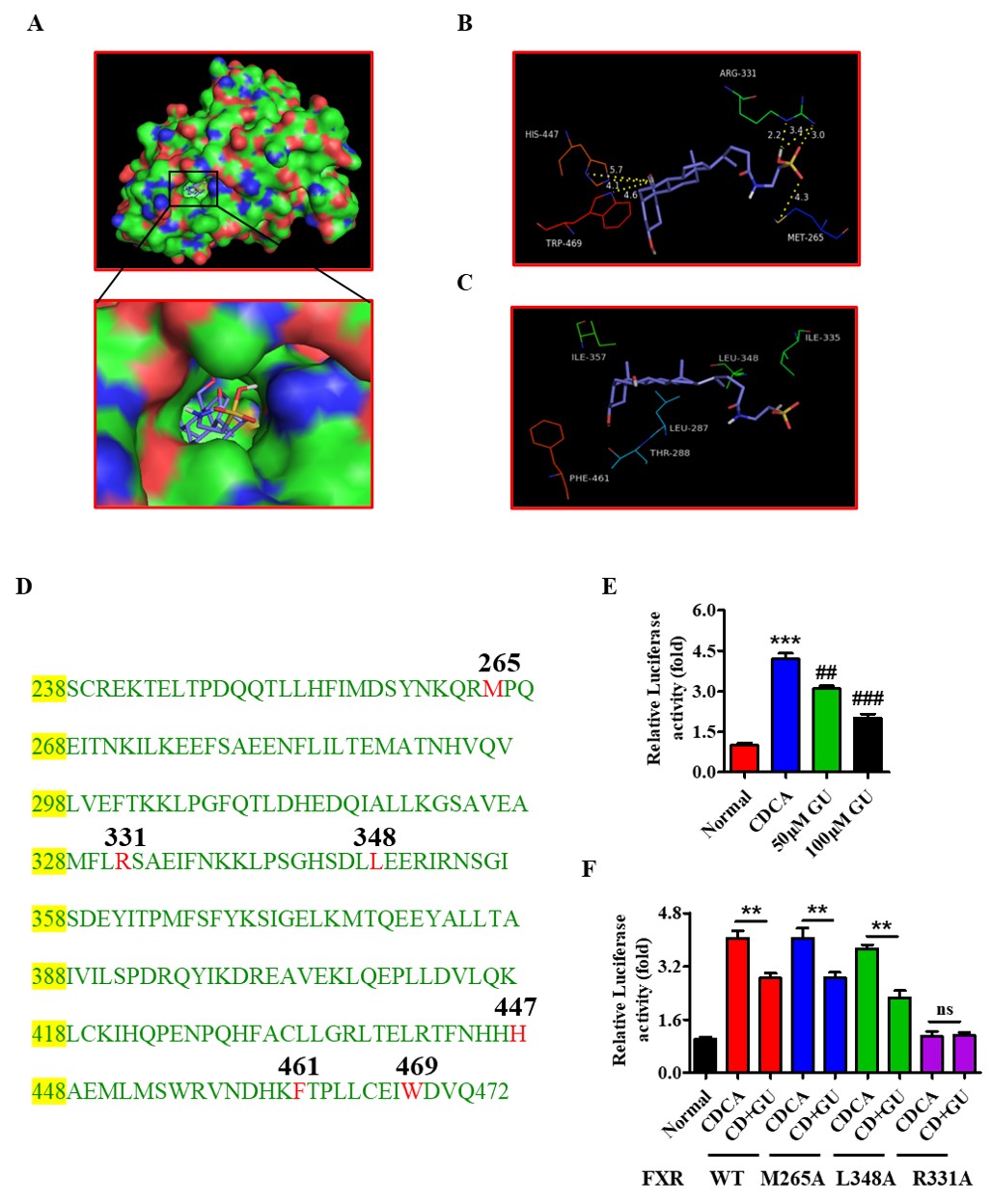

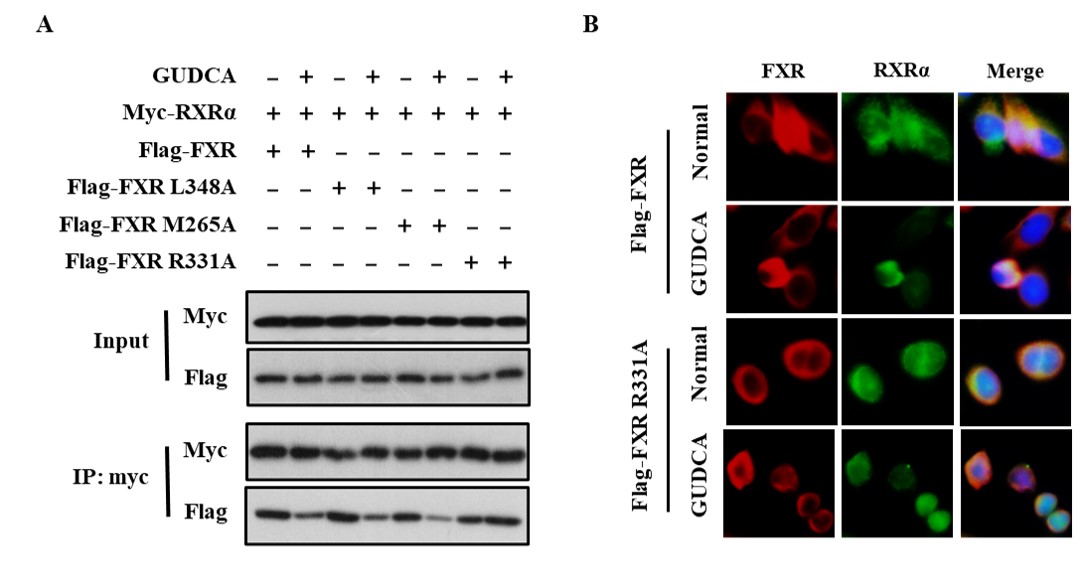

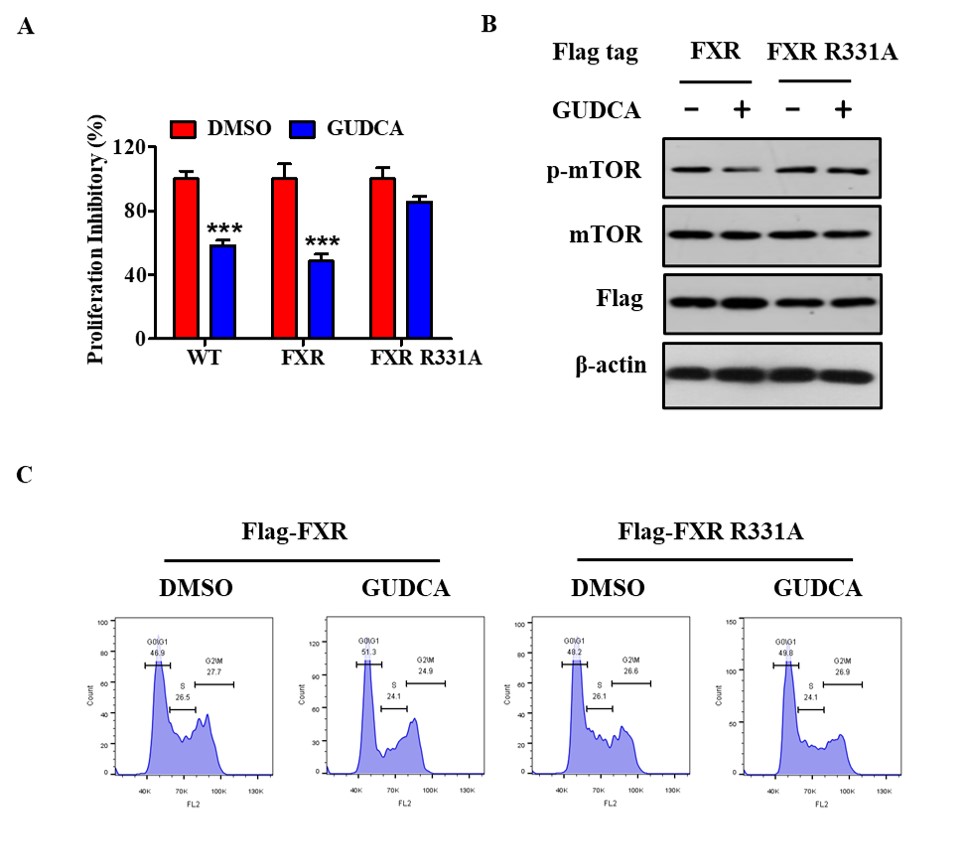

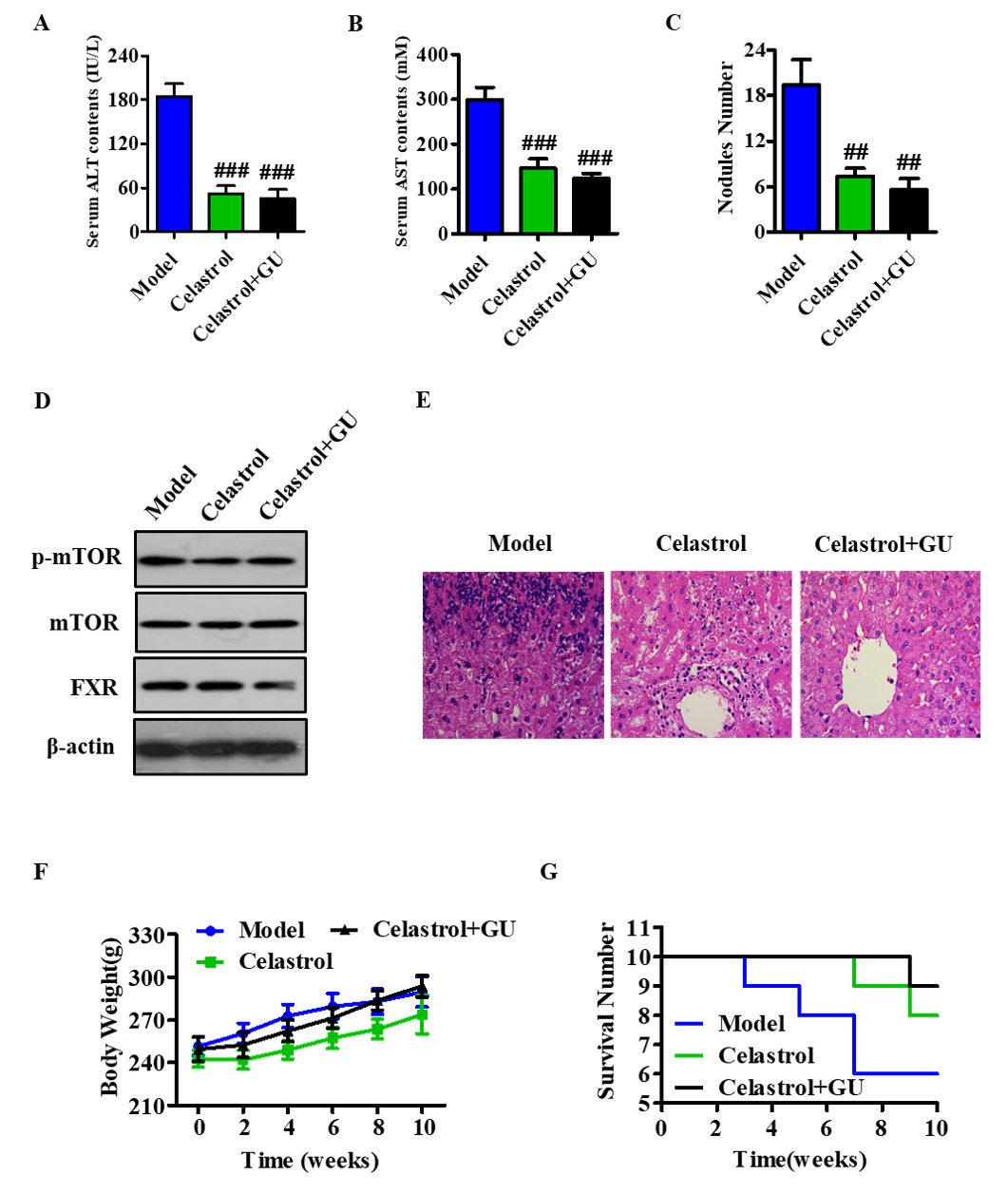

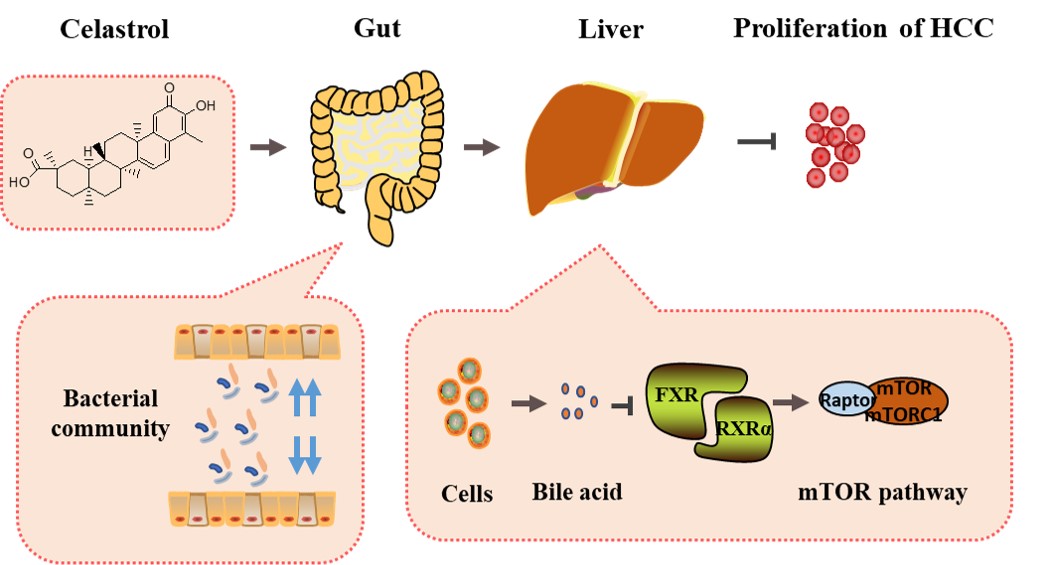

Supplement: Supplementary file 2 [file Table1.DOCX]
